# DRPnet: automated particle picking in cryo-electron micrographs using deep regression

**DOI:** 10.1186/s12859-020-03948-x

**Published:** 2021-02-08

**Authors:** Nguyen Phuoc Nguyen, Ilker Ersoy, Jacob Gotberg, Filiz Bunyak, Tommi A. White

**Affiliations:** 1 Department of Electrical Engineering and Computer Science, University of Missouri, Columbia, MO USA; 2 Institute for Data Science and Informatics, University of Missouri, Columbia, MO USA; 3 Research Computing Support Services, University of Missouri, Columbia, MO USA; 4 Department of Biochemistry, University of Missouri, Columbia, MO USA; 5 Electron Microscopy Core, University of Missouri, Columbia, MO USA

**Keywords:** Convolutional neural network, Regression, Deep learning, Electron microscopy, Image segmentation, Particle picking, CryoEM, Autopicking, Single particle reconstruction, Single particle analysis, 3D reconstruction

## Abstract

**Background:**

Identification and selection of protein particles in cryo-electron micrographs is an important step in single particle analysis. In this study, we developed a deep learning-based particle picking network to automatically detect particle centers from cryoEM micrographs. This is a challenging task due to the nature of cryoEM data, having low signal-to-noise ratios with variable particle sizes, shapes, distributions, grayscale variations as well as other undesirable artifacts.

**Results:**

We propose a double convolutional neural network (CNN) cascade for automated detection of particles in cryo-electron micrographs. This approach, entitled Deep Regression Picker Network or “DRPnet”, is simple but very effective in recognizing different particle sizes, shapes, distributions and grayscale patterns corresponding to 2D views of 3D particles. Particles are detected by the first network, a fully convolutional regression network (FCRN), which maps the particle image to a continuous distance map that acts like a probability density function of particle centers. Particles identified by FCRN are further refined to reduce false particle detections by the second classification CNN. DRPnet’s first CNN pretrained with only a single cryoEM dataset can be used to detect particles from different datasets without retraining. Compared to RELION template-based autopicking, DRPnet results in better particle picking performance with drastically reduced user interactions and processing time. DRPnet also outperforms the state-of-the-art particle picking networks in terms of the supervised detection evaluation metrics recall, precision, and F-measure. To further highlight quality of the picked particle sets, we compute and present additional performance metrics assessing the resulting 3D reconstructions such as number of 2D class averages, efficiency/angular coverage, Rosenthal-Henderson plots and local/global 3D reconstruction resolution.

**Conclusion:**

DRPnet shows greatly improved time-savings to generate an initial particle dataset compared to manual picking, followed by template-based autopicking. Compared to other networks, DRPnet has equivalent or better performance. DRPnet excels on cryoEM datasets that have low contrast or clumped particles. Evaluating other performance metrics, DRPnet is useful for higher resolution 3D reconstructions with decreased particle numbers or unknown symmetry, detecting particles with better angular orientation coverage.

## Background

**Fig. 1:**
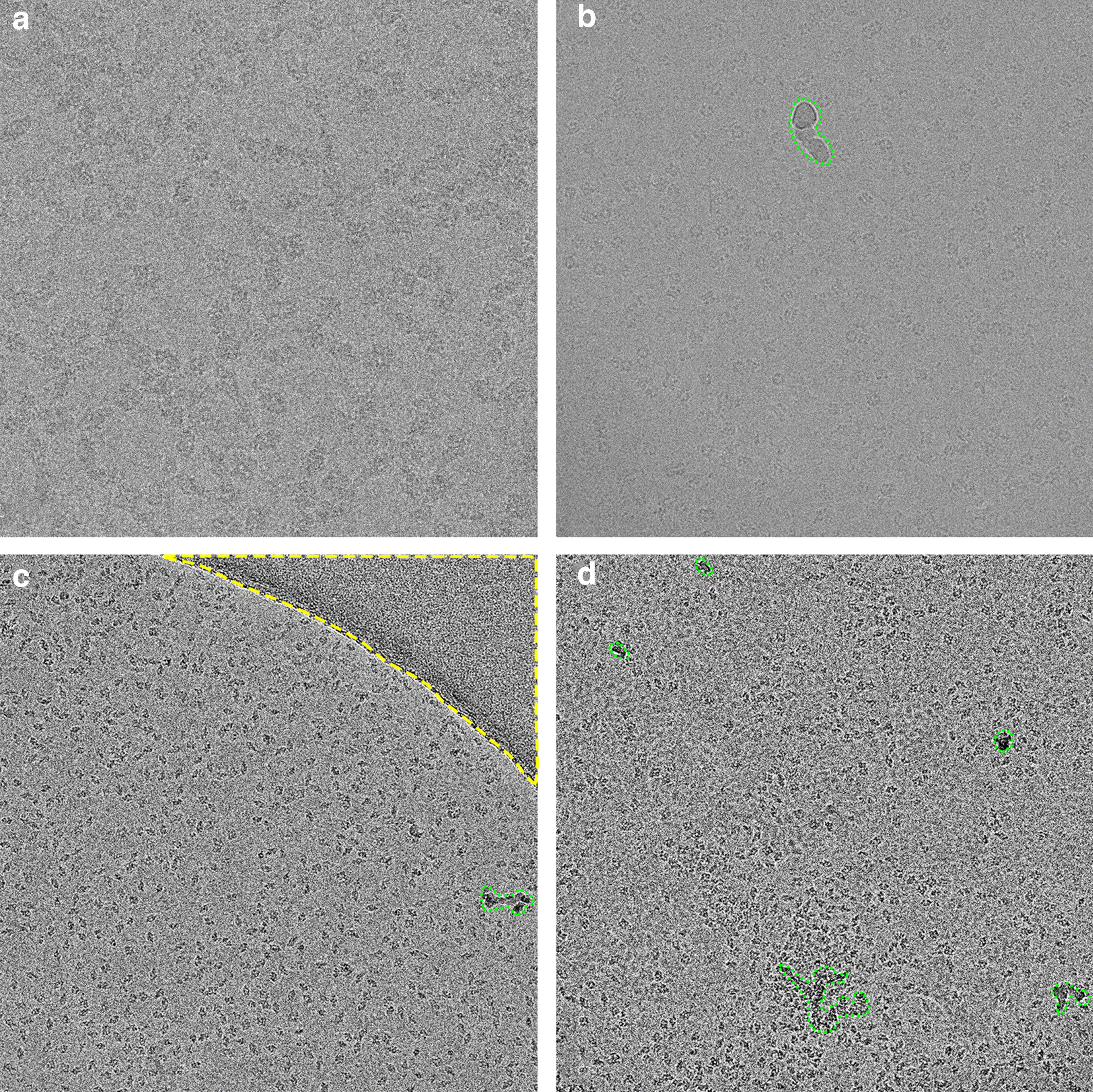
Artifacts and challenges in cryoEM images. **a** Low contrast image (EMPIAR-10061) **b** Green region: non-vitreous ice contamination (EMPIAR-10005) **c** Green region: non-vitreous ice contamination; Yellow region: carbon film (EMPIAR-10017) **d** Green region: non-vitreous ice contamination; Overlapped particles (EMPIAR-10017)

Although high resolution 3D protein structure determination via single particle analysis (also known as single particle reconstruction) using cryo-electron microscopy (cryoEM) is becoming more widely used, it still remains to be a challenging technique because of the resulting noisy and often low contrast micrographs [1, 2]. In these cryoEM experiments, a purified, homogeneous protein is vitreously frozen in a thin film of solution to form a glass-like ice, which is then imaged under cryogenic temperatures ($-\,170\,^\circ \hbox {C}$) in a transmission electron microscope (TEM) [3]. Many cryoEM micrographs are collected, with protein “particles” imaged in different orientations in 2D. Many 2D particle views (also known as “projections”) are collected and used to reconstruct an atomic 3D reconstruction [4] by iterative alignment, classification and averaging. The resulting micrographs are extremely noisy; the low signal is due to a number of causes—microscope-related aberrations, low doses of electron exposure applied to the radiation sensitive protein specimen [5], movement of the specimen upon imaging [6], and the process of high resolution image formation in the TEM [7, 8]. Thus, the protein particles of interest are sometimes challenging to identify in these 2D cryoEM micrographs, especially certain orientations of the particles (see Fig. 1 for examples and artifacts). Several software solutions have been developed to reconstruct 3D protein structures. These solutions require large numbers of particles to accurately estimate the relative angular orientations of these protein particles in 3D, which is then used to create 3D reconstructions of averaged protein particle structures.

One solution, the widely adopted RELION software [9], employs a likelihood-based approach and expectation maximization algorithm to determine each protein particle’s orientation, and then classifies and averages those similarly oriented particles together to generate a high resolution 3D reconstruction of the protein’s structure—or a map representing the Coulombic potential [10]. RELION’s workflow allows users to manually identify and select (“picking”) particles in 2D cryoEM micrographs; these selected particles are then aligned, classified, and averaged to produce 2D class averages. Suitable 2D class averages are manually selected as templates for automated particle selection (“autopicking”) which assesses correlation of image patches to the template particles [11]. We further refer to this as Template Based Autopicking (TBA). After obtaining 2D class average templates from manually picked particles, these software packages rely on two main methods to automatically select particles: template matching by cross-correlation, or pattern recognition by a simple deep neural network. Template matching is sensitive to noise and may suffer from strong bias [11].

Recent advances in machine learning, specifically in deep learning, have led to great improvements in automated biomedical image analysis [12–14]. For microscopic image analysis, [15] many machine learning approaches have been utilized from support vector machines (SVM) to convolutional neural networks (CNN). A recent study of Shin *et al.* [16] has employed deep learning models to learn semantics in MRI scans, and to extract features to detect different organs. Xie [17] proposed a novel deep neural network for robust nucleus localization, where, instead of using a pixel-wise classifier or a regressor, they combined CNN with a nonlinear voting transformation. Inspired by the success of these examples, we sought to apply deep learning methods to cryoEM image analysis, specifically to particle picking, which is the most tedious step in most cryoEM image analysis workflows.

### Machine learning applied to cryoEM particle picking

Numerous software solutions to date have implemented deep learning approaches to decrease time involved in manual particle picking, however a robust solution for a majority cryo-EM datasets is lacking due to the variety of reasons (protein shapes and sizes, grayscale variations, particle distributions and clumping, ice thickness differences, crystalline ice contamination, presences of support films, changes in illumination) as observed in cryoEM datasets (see Fig. 1). We provide a brief overview of some of these networks; however an exhaustive list and subsequent descriptions are not appropriate here.

DeepPicker [18] is one of the earliest fully automated particle picking tools. It slides a window (box) across the micrographs with a default step size of four pixels to collect candidate image patches. Extracted image patches are normalized and fed into a convolutional neural network to determine whether they belong to a qualified class of particles or not. In the fully automated mode, DeepPicker has a pretrained network to pick particles for the first time as positive training samples. The negative samples are randomly selected far from the positive samples using a spatial distance threshold. The positive and negative samples are then used to train a fresh convolutional network to pick the final particles.

Recently, three particle picking packages using deep learning have gained popularity; TOPAZ [19], WARP [20] and crYOLO [21]. Similar to DeepPicker [18], TOPAZ [19] is a deep learning-based particle picking software. It examines micrograph patches and uses non-maxima suppression to select the patches with highest scores as the most likely particle instances. One major difference between TOPAZ and typical deep learning pickers is that TOPAZ can be trained with a small number of positive and unlabeled samples, versus of positive and negative samples. WARP [20], relies on residual neural network (ResNet) architecture [22] that uses skip connections, or shortcuts to jump over some layers for improved performance. WARP was trained with multiple EMPIAR raw and simulated data. As a result, it can pick properly many types of particles and can mask out the artifact regions. WARP also supports network re-training with custom data. crYOLO [21] utilizes a general purpose, single-stage, deep object detector YOLO [23] to detect particles. YOLO detection is based on a fixed-grid regression method that makes it faster compared to other detection networks [24]. crYOLO was trained with 45 datasets and is able to auto-pick particles from previously unseen datasets. Its detection performance was fast, up to five micrographs per second with image dimensions of $1024 \times 1024$ pixels [21] and reached higher accuracy (recall, precision, and AUC) compared to the original YOLO model when working with small objects as particles.

We present below a deep learning algorithm entitled, Deep Regression Picker network (or “DRPnet”), for greatly improving the time taken to manually select particles from cryoEM micrographs. This solution also seeks to improve the accuracy of particle picking using a cascade of convolutional neural networks—the first uses deep regression to identify particles, the second uses a classification network to remove false positives. This network can be used with no training as demonstrated on multiple datasets or with retraining the second classification network for enhanced performance.

DRPnet was trained on one dataset (EMPIAR-10005 TRPV1), which was then used to pick particles on other datasets (EMPIAR-10061 $\beta$-galactosidase, EMPIAR-10017 $\beta$-galactosidase). We compare these DRPnet-picked particles to a similar number of particles generated by RELION’s Template Based Autopicking (using a randomized subset of particles that went into the published 3D reconstruction to simulate a “manually picked” template). Using these DRPnet or TBA particles, we generated 3D reconstructions in RELION. We show that compared to RELION’s TBA, DRPnet-picked particles result in consistently more 2D classes, improved orientation angle distributions, and allows small gains in resolution, especially when symmetry is not imposed. Finally, we compare our solution to other deep learning-based particle-picking networks (WARP, TOPAZ, crYOLO, DeepPicker) in terms of recall, precision and F-measure using both the pretrained networks and after retraining their models. Experimental results with the dataset (EMPIAR-10017, $\beta$-galactosidase) show that DRPnet achieves recall, precision, and F-measures values of 87.7%, 71.1%, and 78.5% respectively. These values are comparable or better than the current state-of-the-art when using the pretrained networks, and DRPnet outperforms the state-of-the-art methods when all the methods are trained with the same dataset and tested on unseen data. DRPnet rivals popular deep-learning cryoEM particle picking algorithms in terms of time and quality, especially compared to manually picked, template-based autopicking, and is freely available on GitHub (https://github.com/emcoregit/DRPnet).

## Methods

**Fig. 2:**
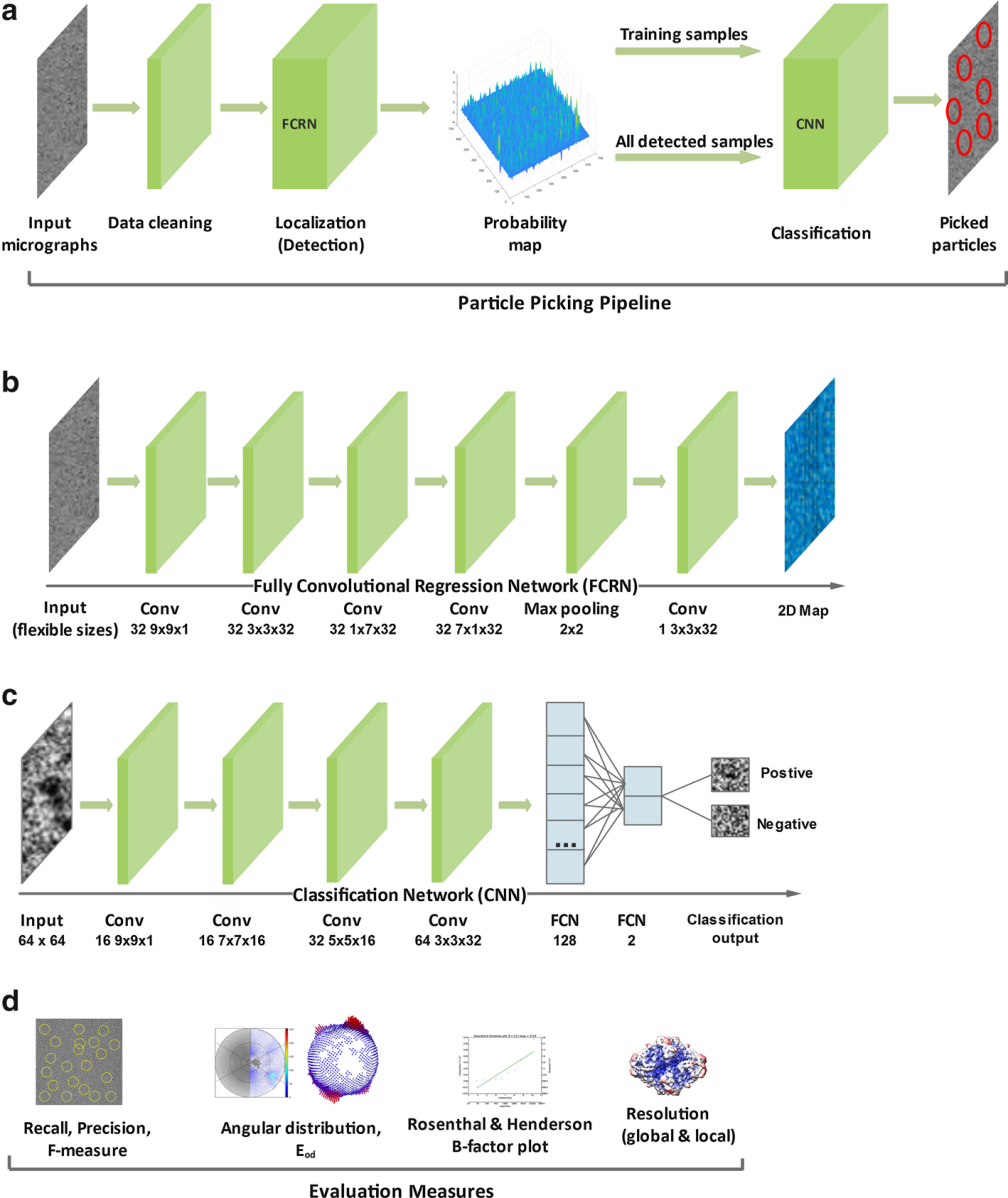
Cascaded architecture of the Deep Regression Picker Network (DRPnet). **a** Overview of the DRPnet particle picking pipeline. Processing stages from input micrographs to picked particles. **b** Architecture of the Fully Convolutional Regression Network (FCRN) used for initial particle detection. **c** Architecture of the Convolutional Neural Network (CNN) used for filtering/refinement of the detections through binary classification. **d** Evaluation metrics used in this study to assess particle picking performance

Inspired by the recent successes of deep learning in object detection [25], we set forth to develop a robust and flexible deep learning-based particle picking system that can handle multiple types of data, various particle sizes and shapes/aspect ratios, and different imaging technologies (i.e. direct detectors) with different defocus ranges. Below, we present our deep learning based particle picking framework, Deep Regression Picker Network (DRPnet) illustrated in Fig. 2. The proposed system works on multiple types of data (tested on three protein datasets) from various detectors, with improved speeds (testing time is approximately $\sim 6$ s/micrograph on Nvidia GTX 1080 GPU with 8GB memory and SSD storage) compared to manual template selection ($\sim 30$ min to select 1000 particles) as implemented by RELION. This RELION implementation includes manually selecting particles, then selection of 2D class averages to generate a template, and finding the autopicking parameters which would provide suitable number of particles—from here on, we will refer to this process as RELION’s template-based autopicking (TBA).

### Deep regression picker network (DRPnet) particle picking pipeline

The particle picking process in DRPnet involves two steps: localization (or detection) and classification (refinement). In classical computer vision, object detection and classification processes often rely on carefully hand-crafted image features and descriptors such as HOG (Histogram of Oriented Gradients) [26], SIFT (Scale-Invariant Feature Transform) [27], or SURF (Speeded-Up Robust Features) [28], etc. that are extracted from image patches. These descriptors are then fed to unsupervised or supervised machine learning models such as SVM (support vector machine), random forests, and neural networks to cluster or to classify those feature vectors. The success of these systems heavily depends on the selected or engineered feature descriptors that may not capture the complexities of the underlying visual patterns unless they can adapt to new patterns.

On the other side, deep learning approaches rely on data itself to learn discriminative features to perform the given task. Convolutional neural networks (CNN), a class of deep learning methods heavily used for image analysis, extract features over multiple convolutional layers with different sizes and different numbers of filters whose coefficients are learned during training. Earlier layers of convolutional networks learn deep features, while later fully connected layers perform the classification or regression tasks. Deep object detection approaches can be coarsely grouped as single-stage and two-stage. Single-stage detectors such as YOLO [29] and SSD [30] rely on regular dense sampling of objects, scales, and aspect ratios, and perform detection and classification in one step. Two-stage detectors such as FasterRCNN [31] and Mask-RCNN [32] first produce a sparse set of candidates (region proposals). These candidates are then classified into a number of classes, foreground versus background in our case. Faster-RCNN and Mask-RCNN have shown great performance in detection and classification tasks on image datasets such as Pascal VOC [33] and MS COCO [34]. However, these models were originally designed to work with larger objects with rich color, texture, and shape features. The features of the benchmark datasets used for training these standard detection networks are quite different when compared to features of cryo-electron micrographs . Unlike objects in these benchmark datasets, cryo-electron micrographs (see Fig. 1) are grayscale, low contrast (Fig. 1a), and may contain noise and artifacts caused by factors such as surface ice contamination (Fig. 1b), support-film edges (Fig. 1c), or overlapped particles (Fig. 1d).

In order to overcome the aforementioned issues, we propose the Deep Regression Picker Network (“DRPnet”, Fig. 2), which is based on the blob detection concept [35]. To better support the ability of localization and classification, we have developed a two-stage particle picking pipeline (Fig. 2a). The first stage consists of a fully convolutional regression network (FCRN) designed for particle candidate localization/detection (Fig. 2b); while the second stage consists of a classification convolutional neural network (CNN) designed to refine the detections (Fig. 2c). While the second stage classification network is a supervised model to classify true versus false positives, training data for this task was collected in an unsupervised manner by using the first stage regression network’s measure of confidence. High and low confidence levels were used as indications for true positives and true negatives respectively. The initial network was trained using the EMPIAR-10005 dataset [36]. This scheme allows DRPnet to operate on other datasets automatically from detection through classification. After the initial training with one dataset, the proposed DRPnet system was tested with multiple particles having various sizes, shapes, and distributions from different datasets. We tested 7 datasets from two modalities, (cryoEM versus negative stain) with relatively different particle sizes/pixels without the need for retraining demonstrating the robustness of DRPnet.

### Data preprocessing

In order to ensure optimal performance by both networks, the proposed particle picking pipeline also includes a micrograph preprocessing step to enhance contrast and to correct transmission/illumination artifacts. Illumination (beam centering) and/or electron transmission (transmitted electron signal) can misguide DRPnet’s detection because particle picking significantly relies on intensity of grayscale images. To obtain the best DRPnet detection performance, it was necessary to reduce the influence of illumination/transmission variations. We compute local averages of illumination by applying a low-pass filter with a very large kernel. The corrected intensity value of each pixel is obtained by subtracting local and adding global intensity averages, as in Eq. 1:

1 $$\begin{aligned} I_{new} = I - I_{\sigma } + \frac{1}{n}\sum (I_{\sigma }) \end{aligned}$$

where *I* is the original image, *n* is the number of pixels in the image, and $I_{\sigma }$ is image smoothed with a Gaussian filter of sigma $\sigma$, set heuristically according to particle size.

### Deep learning

#### Stage 1: Fully convolutional regression network (FCRN)

**Fig. 3:**
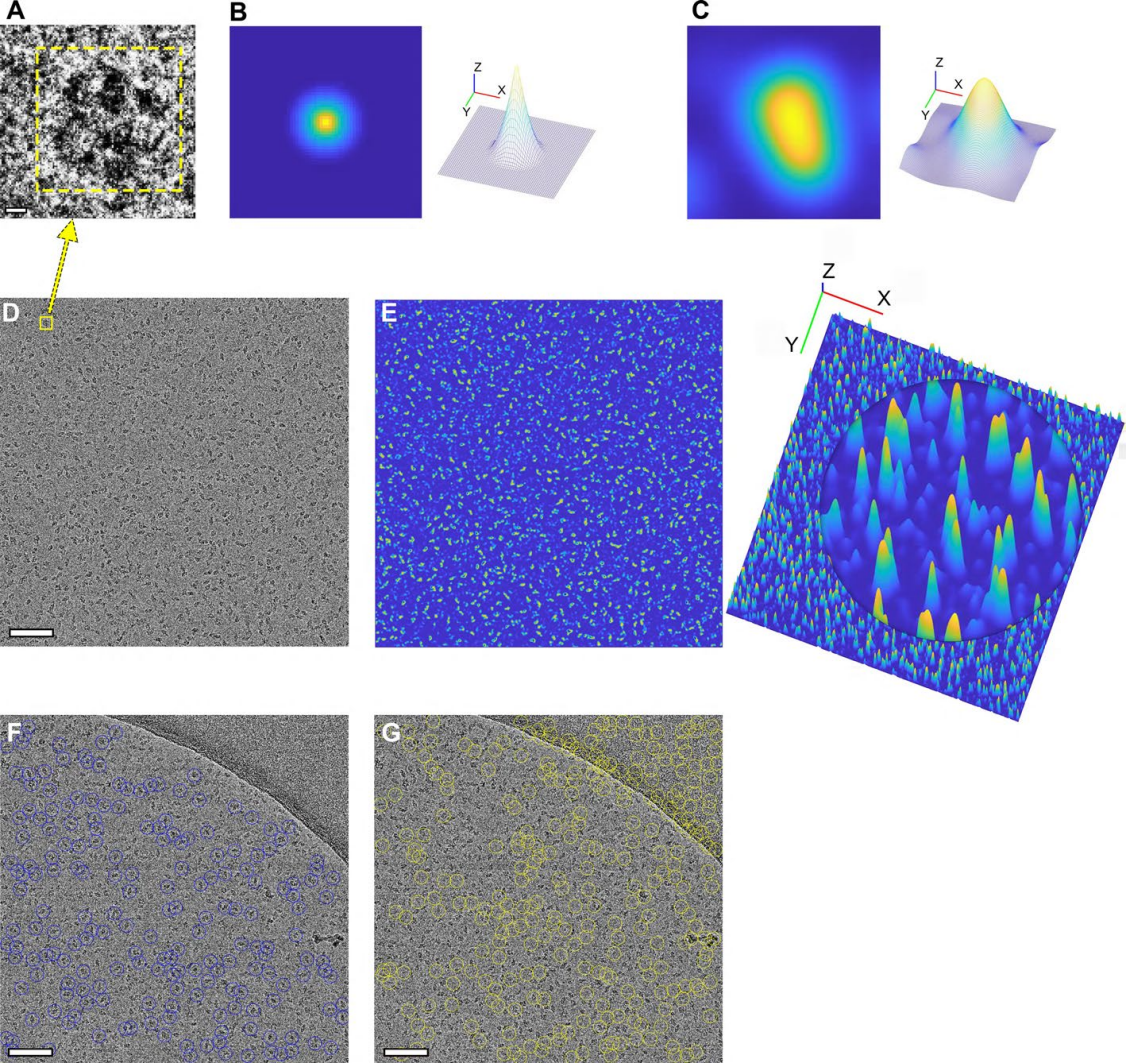
Sample training and test images for the proposed DRPnet from selected cryoEM micrographs. **a** A magnified image patch of a TPRV1 particle (scale is 50 Å). **b** Corresponding ground truth training label obtained by applying the distance transform to the binary particle mask, with blue and yellow indicating lower and higher distance values, respectively. **c** Smoothed 2D particle prediction map corresponding to the output of the fully convolutional regression network shown in Fig. 2b for a single particle (left) and its 3D visualization (right). **d** Sample cryoEM micrograph input into DRPnet (scale is 885 Å). Yellow box represents the particle shown in **a**. **e** Particle prediction map from C computed by DRPnet for the entire cryoEM micrograph (left), and its 3D visualization with circled area showing a magnified view of the local maxima (right). **f**, **g** Positive (**f**, blue circles) and negative (**g**, yellow circles) particles used to train the classification network shown in Fig. 2c. Positive samples represent true particles, negative samples represent false detections. These positive and negative training samples are selected in an unsupervised way using the prediction confidence values from the fully convolutional regression network depicted in Fig. 2b, with high or low confidence particles corresponding to positive or negative training samples, respectively (scale is 885 Å)

To address the challenges of automated particle picking, we have treated this task as a blob detection problem - where each particle is assumed to be a roughly convex blob with texture different than the background (Figs. 2b and 3a). Additionally, we assume that the dimensions and shapes of particles are relatively similar. Given a rough size estimate, our goal is to locate particle centers. Our proposed model is a Fully Convolutional Regression Network (FCRN), trained to predict particle centers by producing a likelihood map where local maxima correspond to the particle centers (Fig. 3). Given the ground truth particle centers (as provided by the datasets in EMPIAR) along with a particle size estimate, first, binary particle-versus-background masks are produced for the cryo electron micrographs. Then, distance transforms of those binary masks are used as training labels for the proposed FCRN model (Fig. 3a). The distance transform of a binary image is a grayscale map where each pixel’s value represents the distance to the closest boundary. This is calculated at each pixel by the distance to the nearest background pixel. Distance transform values for the foreground regions (or the particles) increase from a particle’s boundary towards the particle’s center, reaching the local maxima at the particle’s center (Fig. 3b), and the distance transform for the background pixels are set to zero (Fig. 3b, left). The distance transform of the particle mask produces an estimate of the particle center probability map (Fig. 3b, right). This continuous regression model (FCRN), trained with the particle distance transforms, is then used to localize particle centers. This approach is different than deep regression used to estimate object coordinates (i.e. object bounding box coordinates) that is adopted by many deep object detectors such as FasterRCNN [31] or YOLO [29]. Unlike traditional deep object detectors that regress to a discrete set of coordinates, the proposed network learns to regress to a continuous distance map that acts like a probability density function of particle centers. This regression approach has considerable advantages over direct regression to particle center coordinates: (1) the continuous mapping captures information on not only particle centers, but also on particle shapes; (2) learning to approximate distance transform implicitly regularizes the output making the continuous representation of the particle centers more robust to noise compared to discrete coordinates (which is very important for processing of extremely noisy cryo-EM images). Furthermore, because of its continuous nature, this model also enables localization of an individual particle within a dense cluster, and ensures better scale adaptation, which allows detection of different sized particles without need for retraining the network.

The proposed FCRN has seven layers (Fig. 2b); one input layer, five convolutional layers, and one max-pooling layer [37]. With its simple structure, our approach has smaller computational cost and training data needs, compared to more complex deep learning models such as Fast-RCNN. The proposed network was implemented using the Matlab deep learning toolbox. The network was trained with image patches (Fig. 3a) from raw micrographs and their associated training labels, centered and cropped on particle regions (Fig. 3b); their prediction is shown in (Fig. 3c). These image patches are of the same size as the FCRN input layer. The number of patches extracted from each image is equal to the number of selected particles in those images. We used Adam optimizer [38] to train this network. Since our network is built without fully connected layers, it does not require fixed size input images. In the testing stage, we directly applied the trained FCRN model to different-sized input images (for example, an entire 2D cryoEM micrograph, Fig. 3d) to predict associated particle center probability maps (Fig. 3e, left). Figure 3e(right) shows the associated 3D visualization.

#### Stage 2: Classification network

The first network (Fig. 2b) outputs candidate particle locations, but suffers from false detection. The second convolutional neural network (CNN) refines these particle candidates using a learned keep-or-reject candidate process (Fig. 2c). We extracted two subsets from the particles detected by the first FCRN network, and trained a second two-class classification network using stochastic gradient descent optimization [37]. Positive training samples were particles detected with high confidence, then further refined by removing those with low standard deviation of intensity. Negative training samples were particles with low probability scores in the detection map, as well as particles which had a low standard deviation of intensity. Negative particles included patches of background, carbon edges, ice contaminated regions, and weak patterns. Using the second convolutional network, these particles are classified into positive particles (Fig. 3f) and negative particles (Fig. 3g). This second classification network learns texture patterns of true particles versus false positives caused by various artifacts, and provides a refinement to the particle detection process in an efficient, unsupervised manner. This network has four convolutional layers each followed by a max-pooling layer [37] for feature extraction and two fully connected layers [37] to decide whether to pick or discard a particle patch using the learned features.

DRPnet that consists of this cascade of two networks was successfully implemented and tested using Matlab 2018b Image Processing Toolbox, Computer Vision Toolbox, and Deep Learning Toolbox. DRPnet is freely available on GitHub at https://github.com/emcoregit/DRPnet.

### 3D reconstruction pipeline

Taking cryoEM datasets available in EMPIAR [39], we utilized both DRPnet-particle picking and RELION (v3.0) TBA to generate datasets with similar numbers of particles. For each EMPIAR dataset utilized, RELION requires generation of a manually picked particle set, usually around 1000 particles total. Particles used in our RELION comparison results were generated by randomly selecting a subset (${\sim }$ 1000) of particles deposited in EMPIAR that contributed to the final, refined high resolution 3D reconstructions previously published. These randomly selected particles can be considered the “manually picked” particles. These particles were then processed through the standard RELION pipeline (template generation by 2D classification). DRPnet particles were generated as described before. Particles that have a distance to input image edge less than the box size (as defined by the user in DRPnet) are filtered to avoid incomplete detection of particles, Both DRPnet particles and the RELION TBA particles were then processed through RELION 2D classification to identify good 2D class averages (see Fig. 3 in chapter 6 of Methods in Enzymology [40]). Then both DRPnet and RELION TBA 2D class averages were further 3D classified (allowing 5 possible classes) using the corresponding deposited, low-resolution (60 Å) filtered 3D reconstruction as the initial model. All good 3D classes were selected and refined to generate the final 3D reconstruction.

### Performance metrics

We used multiple metrics to measure the particle picking performance of the proposed DRPnet pipeline (Fig. 2d). Performance metrics have been poorly defined to assess the output provided by the aforementioned, previously-developed particle picking tools. We sought to provide a battery of metrics which may provide insights into particle picking performance. Most tools involve a manual/visual inspection step of the 2D class averages computed using the picked particles and the resultant 3D reconstructed map. In addition to manual/visual inspection, we have proposed five quantitative particle picking evaluation measures:(1) recall, precision, F-measure, (2) global resolution, (3) local resolution, (4) angular distribution, and (5) Rosenthal and Henderson B-factor plots that are described below.

#### Recall, precision, F-measure

Given a dataset with ground truth particle locations, detection performance can be quantitatively evaluated using three supervised metrics, recall, precision, and F-measure, as defined below:

2 $$\begin{aligned} Recall= & {} \frac{TP}{TP+FN} \quad \quad \quad Precision=\frac{TP}{TP+FP} \end{aligned}$$

3 $$\begin{aligned} F= & {} 2\times \frac{Precision \times Recall}{Precision+Recall} \end{aligned}$$

where TP, FP, FN refer to true positives, false positives, and false negatives, respectively. These metrics are used to compare the particle detection performance of DRPnet to other state-of-the-art deep-learning based particle picking networks. Also refer to Table 1 for the prediction outcomes and confusion matrix.

**Table 1:** Prediction outcomes and confusion matrix

	Actual positive	Actual negative
Positive prediction	TP	FP
Negative prediction	TN	FN

#### 3D reconstruction global resolution

An evaluation metric we applied to evaluate the performance of particle picking is the final resolution of the 3D reconstruction. In cryo-electron microscopy, we rely on the concept of signal-to-noise ratio (SNR), and measure the internal consistency of the 3D reconstructions generated by autopicked particles (of DRPnet or RELION TBA). Taking advantage of the Fourier transform, correlation between two 3D reconstructions can be represented by a product in Fourier space, and then that product is split into shells by radial frequency to compute Fourier Shell Correlation (FSC; Eq. 4) [9, 41–43].

4 $$\begin{aligned} FSC(k,\Delta k) = \frac{{{\mathop {\mathrm{{Real}}}\nolimits } \left( {\sum \nolimits _{(k,\Delta k)} {{F_1}(K){F_2}(K)} } \right) }}{{{{\left( {\sum \nolimits _{(k,\Delta k)} {{{\left| {{F_1}(K)} \right| }^2}{{\left| {{F_2}(K)} \right| }^2}} } \right) }^{1/2}}}} \end{aligned}$$

where *K* represents spatial frequency vector, $k = |K|$, is the magnitude of spatial frequency, $\Delta K$ denotes ring width or shell thickness, and $F_1(K), F_2(K)$ are the Fourier transforms of the two half set reconstructions.

In the 3D reconstruction process, a plot of FSC versus spatial frequency shows that FSC falls off when spatial frequency increases, and the connection between FSC and Spectral Signal to Noise Ratio (SSNR) is explained by [44] as following:

5 $$\begin{aligned} SSNR=\frac{FSC}{1-FSC}, \quad FSC=\frac{SSNR}{SSNR+1} \end{aligned}$$

FSC is a biased estimate of SSNR. For a large number of images variance of SSNR is equal to variance of FSC, and the bias is negligible. When FSC is calculated for a data set which is split into two halves, the relationship is:

6 $$\begin{aligned} SSNR=\frac{2FSC}{1-FSC} \end{aligned}$$

By definition, FSC indicates the consistency of the two 3D reconstructions from particle data. As its relationship with SSNR, FSC is used to identify the resolution of 3D reconstruction. A specific level of FSC correlates to a spatial frequency, and that frequency has a unit of 1/Angstroms (1/Å) which is the inverse of the second measured metric, resolution [42]. To avoid overfitting, those two 3D reconstructions should be generated independently from two halves of data. RELION’s 3D refinement uses 0.143 as a cutoff level between signal and noise to evaluate resolution, and this level is also referred as the gold standard. [9]

7 $$\begin{aligned} FSC = \frac{{\sum {(S + {N_1})(S + {N_2})} }}{{\sum {({S^2} + 2SN + {N^2})} }} \end{aligned}$$

where *S* denotes the signal and $N = {N_1} = {N_2}$ are noise in half datasets.

#### 3D reconstruction local resolution

The resolution estimated by FSC curve is a global evaluation of the 3D single particle reconstruction. To analyze resolution variations in different 3D map regions, Kucukelbir [45] proposed a definition of local resolution. For each voxel, the local resolution is the wavelength of the highest local spatial frequency that is statistically above the noise. The noise level is identified by taking two halves of gold standard 3D map as input to compute both the mean, representing the signal, and the difference between them, representing the noise, as implemented in the software package, ResMap [45]. Instead of using ResMap, we applied RELION’s implementation to perform local resolution analysis in our experiment. RELION utilizes a soft spherical mask moved around the entire 3D map to estimate local resolution, which we used as another metric to evaluate 3D reconstructions using DRPnet-picked versus RELION TBA particles.

#### Angular distribution

Besides the metrics above, we analyzed the angular distributions recorded in RELION .star files of 3D refinement jobs to understand the differences in orientation of particles picked by DRPnet and RELION TBA. Those angular distributions showed the effect of orientation on resolution and clarified the resolution difference in maps obtained by DRPnet and RELION TBA. We visualized the angular distribution by 2D Hammer projection scatter plots of angular coverage [46]. These scatter plots are equivalent to the bild files of RELION’s 3D refinement jobs, and they include the same scale color bars to compare between DRPnet and RELION TBA. Furthermore, we employed efficiency number E$_{\text {od}}$ [47] to confirm the angular distributions’ effect on structure’s resolution. By using corresponding point spread function, Naydenova and Russo [47] computed E$_{\text {od}}$ to assess how the angular distribution contributes to the reconstruction results. An E$_{\text {od}}$ value of 0.8 to 1 indicates a good orientation distribution and uniform Fourier space coverage. If the E$_{\text {od}}$ is lower than 0.5, the lack of particles in angular distribution will cause elongation artifacts in the 3D reconstruction (for example, elongation in an axis that is missing the views).

#### Rosenthal and Henderson B-factor plots

As originally proposed by Rosenthal and Henderson [48], plotting of the inverse-squared resolution as a function of the number of particles allows us to compare numbers of particles picked with DRPnet or RELION and the effect of particle numbers on 3D reconstruction resolution [49]. We used Rosenthal & Henderson B-factor plots as another metric to evaluate the convergence of the 3D reconstructions using DRPnet-picked versus RELION TBA.

## Results

### Datasets

**Fig. 4:**
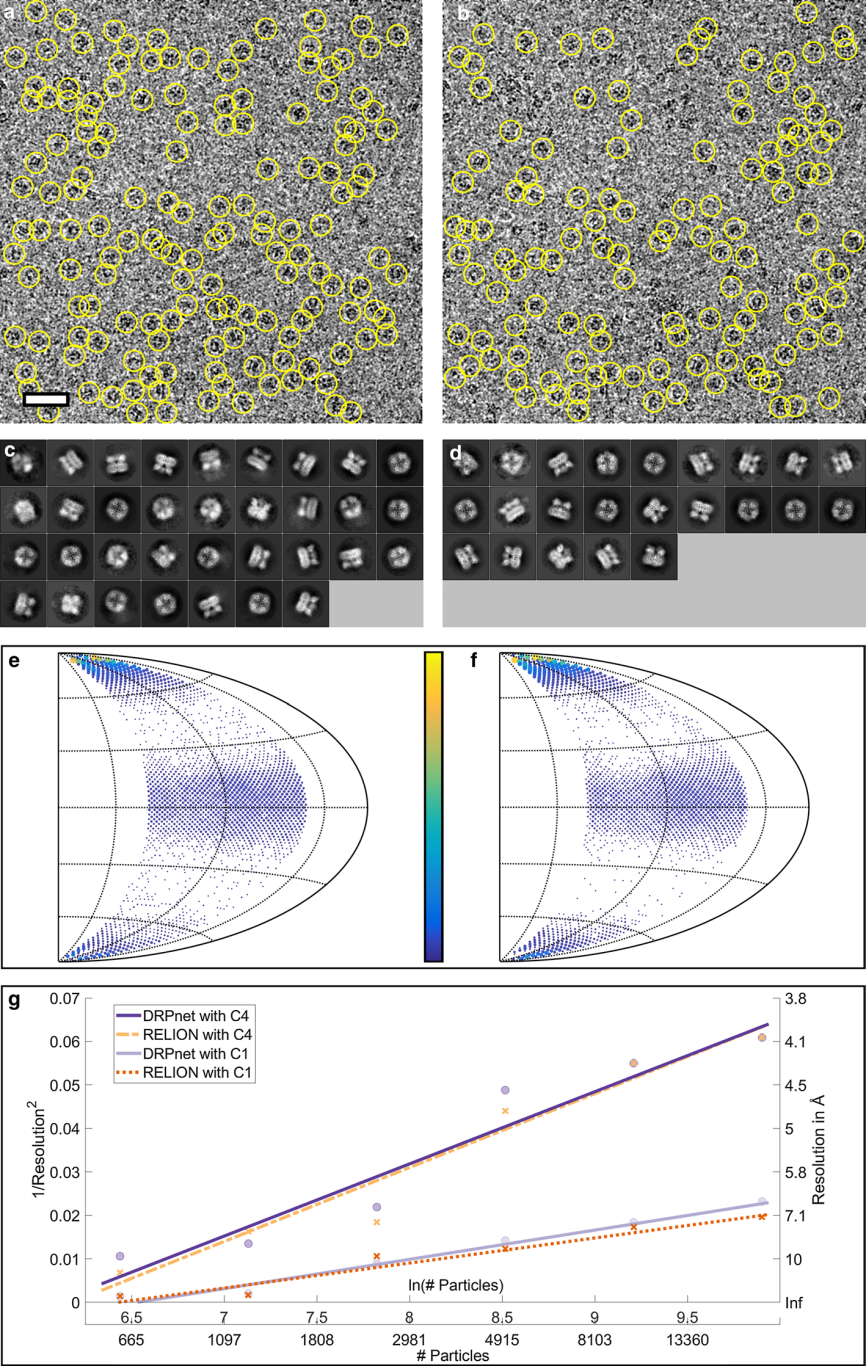
Particle picking results on EMPIAR-10005 (TRPV1) dataset. A representative cryoEM micrograph and picked particles (yellow circles) by DRPnet (**a**) and RELION TBA (**b**) (scale is 500 Å). All picked particles were classified using RELION to select quality 2D class averages as displayed for DRPnet (**c**) and RELION (**d**). The angular distributions were determined during 3D reconstruction and are displayed in angular coverage plots for DRPnet (**e**) and RELION (**f**). The range of the color bar is from 1 view (blue) to 600 views (yellow). Note, better angular coverage is delineated by increased views for DRPnet-picked particles. (**g**) Rosenthal and Henderson B-factor plot shows the relationship between number of particles and the global resolution of the 3D reconstruction

**Fig. 5:**
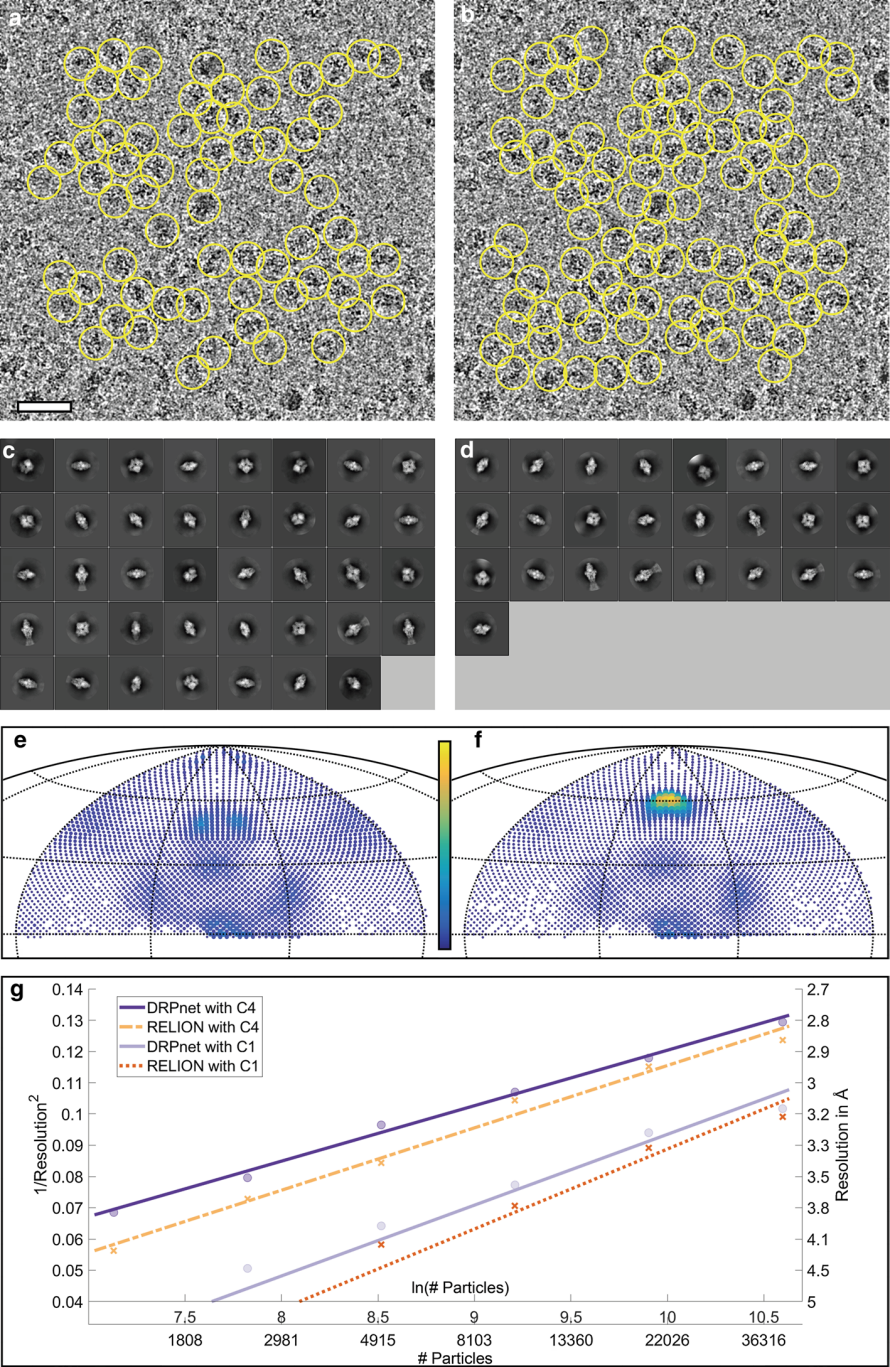
Particle picking results on EMPIAR-10061 ($\beta$-galactosidase): A representative cryoEM micrograph and picked particles (yellow circles) by DRPnet (**a**) and RELION (**b**) (scale is 300 Å). All picked particles were classified using RELION to select quality 2D class averages as displayed for DRPnet (**c**) and RELION (**d**). The angular distributions were determined during 3D reconstruction and are displayed in angular coverage plots for DRPnet (**e**) and RELION (**f**). The range of the color bar is from 1 view (blue) to 700 views (yellow). Note, better angular coverage is delineated by increased views for DRPnet-picked particles. (**g**) Rosenthal and Henderson B-factor plot shows the relationship between number of particles and the global resolution of the 3D reconstruction

**Fig. 6:**
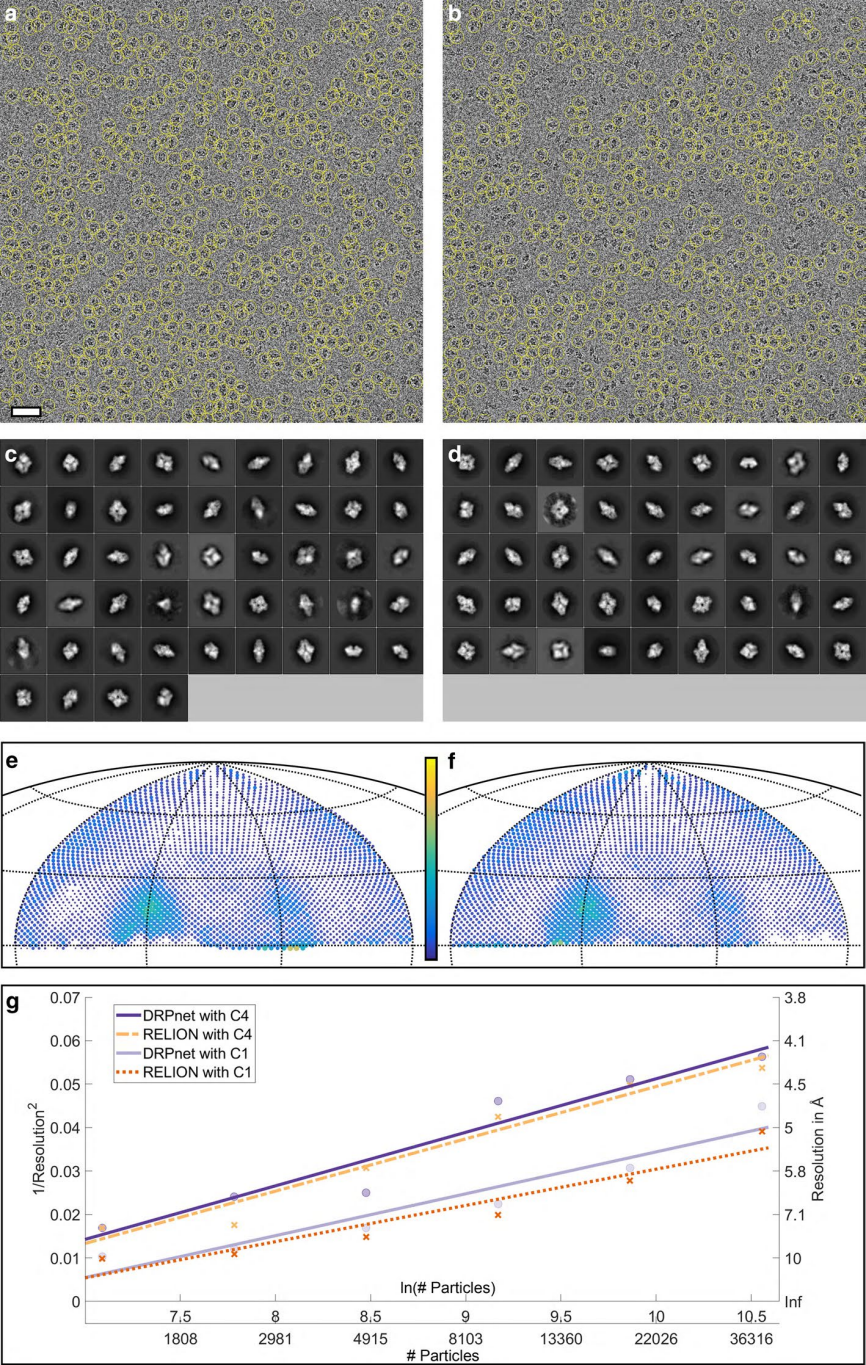
Particle picking results on EMPIAR-10017 ($\beta$-galactosidase): A representative cryoEM micrograph and picked particles (yellow circles) by DRPnet (**a**) and RELION (**b**) (scale is 500 Å). All picked particles were classified using RELION to select good 2D class averages as shown for DRPnet (**c**) and RELION (**d**). The angular distributions were determined during 3D reconstruction and are displayed in angular coverage plots for DRPnet (**e**) and RELION (**f**). The range of the color bar is from 1 view (blue) to 200 views (yellow). Note, better angular coverage is delineated by increased views for DRPnet-picked particles. (**g**) Rosenthal and Henderson B-factor plot shows the relationship between number of particles and the global resolution of the 3D reconstruction

To demonstrate our network’s ability to pick multiple types of particles, we prepared a pretrained DRPnet model from one cryoEM dataset (TRPV1, EMPIAR-10005 [36]) and tested it on other datasets ($\beta$-galactosidase, EMPIAR-10017 [11], and $\beta$-galactosidase, EMPIAR-10061 [50]). These cryoEM datasets were selected because of the instrumentation used to collect the data and the existence of a ground truth. They were acquired on a cryo-transmission electron microscope at 300 kV accelerating voltage using either a FEI Titan Krios (EMPIAR-10005 and EMPIAR-10061) or on a FEI Tecnai F30 Polara (EMPIAR-10017). The direct detector, or camera, differed for each dataset. Please see Table 2 for a summary of the experimental differences between these cryoEM datasets. The particles picked from these datasets were taken through the RELION 3D reconstruction pipeline and results presented in Figs. 3, 4, 5 and 6. Additional experiments were also performed to show the effectiveness of DRPnet picking on particles of various size, shapes, and embedded in different medias and are shown in the supplementary materials. DRPnet particle picking was performed on in house negatively stained small particles including the apo-form of aldehyde dehydrogenase 7A1 [51] (Additional file 1: Figure S3 A-B) and a self-associating Fab fragment, OKT3 (Additional file 1: Figure S3 C-D). Also included is DRPnet particle picking results (Additional file 1: Figure S4 A-D) of the larger T20S proteasome (EMPIAR-10025 [52]) having different particle top and side view shapes.

**Table 2:** Experimental datasets from EMPIAR used for DRPnet testing

Dataset	Voltage (kV)	Camera	Defocus range ($\upmu$m)	# Total images	# Test images	# Picked particles
EMPIAR-10005 (TRPV1)	300	Gatan K2	1.5–3.5	871	661 (subset)	60,000
EMPIAR-10017 ($\beta$-gal)	300	FEI Falcon 2	1.0–3.0	84	84 (full set)	50,000
EMPIAR-10061 ($\beta$-gal)	300	Gatan K2 Summit GIF K2	0.6–3.0	1538	661 (subset)	60,000

### Network training on the EMPIAR-10005 (TRPV1) dataset

The first network of the proposed DRPnet cascade is FCRN (Fig. 2b) that is responsible for particle candidate detection. We have trained the FCRN network with a subset of the TRPV1 dataset (EMPIAR-10005), using 50 TRPV1 micrographs and 9751 ground truth particles extracted from the deposited coordinates used for the high resolution 3D reconstruction [36]. Image dimensions for these cryoEM micrographs were $3710\times 3710$ pixels, pixel size was 1.2156 Å and the TRPV1 particles are 100 Å x 110 Å x 110 Å. We scaled down the images by $3\times$ and used a box size of $64\times 64$ pixels to extract particle patches. Using the deposited particle coordinates, first binary particle masks were generated, then distance transform of those masks were calculated (Fig. 3b) and used to train the FCRN network. The second network of the proposed DRPnet cascade, a classification CNN (Fig. 2c), was trained to detect positive or negative training samples (Fig. 3g, h) using the output of the first FCRN network.

### Network testing on EMPIAR-deposited datasets

#### EMPIAR-10005 (TRPV1) testing

In order to validate the pretrained DRPnet model described above, we tested it on a set of 661 TRPV1 micrographs collected on a Gatan K2 direct detector. TRPV1 micrographs have low contrast, and some micrographs do not contain any particles. To detect the particle candidates, we set the first network (FCRN) parameters at normal detection levels ($\hbox {sigma} = 9$, $\hbox {threshold} = 2\times 5$). Positive and negatives training samples for the second network were selected using the parameter sets $\hbox {sigma} = 9$, $\hbox {threshold} = 3\times 5$ and $\hbox {sigma} = 9$, $\hbox {threshold} = 1\times 5$ respectively. As a result of this process, DRPnet picked 61,282 particles. In order to pick similar numbers of particles, we set the parameters of the RELION TBA to $\hbox {sigma} = 1$ and $\hbox {threshold} = 0.475$.

Picking similar particle quantities ensures a fair comparison between DRPnet and RELION TBA. As has been seen from the Rosenthal-Henderson plots [48] particle quantity effects final 3D reconstruction resolution, but limited by the Nyquist frequency of the data or the flexibility of the protein particle. For each image, the number of true particles is fixed while the number of picked particles varies depending on the threshold. If the set threshold is high, the picking program is likely not to detect all true particles. Conversely, if the set threshold is low, the picking program will pick more false positives (noisy artifacts such as ice contamination, carbon edges, or overlapped particles see Fig. 3). These two cases degrade the quality of 2D image patches thus consequently the resolution of 3D reconstruction. It is necessary to clarify the definition of threshold as used by RELION and how it differs in DRPnet. RELION’s thresholding uses cross-correlation to compare image patches to a set of particle templates. This threshold is used to judge similarity of image patches to the particle template set. DRPnet uses distance transform map as a probability density function of particle centers. In DRPnet, threshold is used to determine if there is enough evidence for a particle at a particular location utilizing the distance transform map.

The template-matching based particle picking function in RELION requires manual picking of about a thousand particles by expert users. The process is not only time consuming but also potentially subjective and biased. Our goal is not to improve performance against expert selected template-based picking, rather to achieve comparable results in a fully automated fashion that is faster and unbiased.

Given these parameters, RELION TBA picked 61,599 particles To perform the subsequent jobs, we extracted particles with box size $200\times 200$ pixels without image scaling. Taking both sets of picked particles forward through the RELION 3.0 pipeline, we selected 23,147 good particles picked by DRPnet and 22,830 good particles picked by RELION TBA to generate the 3D reconstruction.

**Fig. 7:**
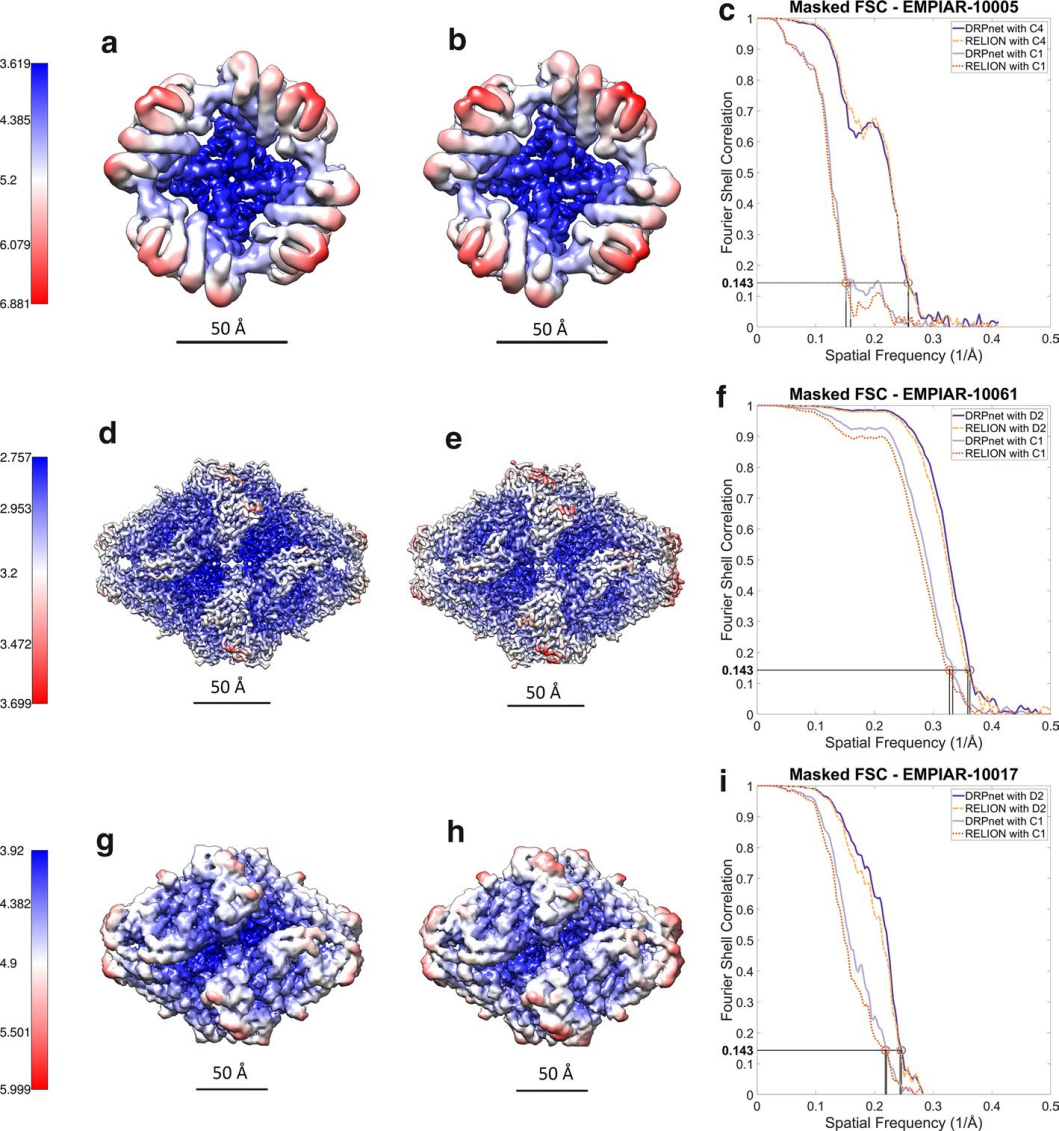
Single particle analysis 3D reconstruction outcomes. Left and middle columns compare the superimposed 3D reconstructions rendered as isosurfaces colored to represent local resolution variations generated from DRPnet-picked (**a**, **d**, **g**) or RELION template-based picked particles (**b**, **e**, **h**), respectively. The 3D reconstruction isosurfaces are colored such that higher resolution regions are colored in blue, and red represents less well-resolved regions (see color scale on left). The right column (**c**, **f**, **i**) contains corresponding masked Fourier Shell Correlation curves for 3D reconstructions, performed with or without symmetry, using DRPnet-picked or RELION TBA, with horizontal line indicating the gold standard (0.143) FSC cutoff. The top row shows 3D reconstructions from EMPIAR-10005 (TRPV1) , the middle row from EMPIAR-10061 ($\beta$-galactosidase) and last row from EMPIAR-10017 ($\beta$-galactosidase)

Figure 4 presents detailed evaluation of particle picking performance by the proposed DRPnet network versus the RELION TBA on the EMPIAR-10005 (TRPV1) dataset. Figure 4a, b shows picked particles on a sample micrograph. As discussed in the “Methods” section, it is observed that DRPnet can successfully pick particles in dense groups, within close proximity of each other. When fed to the RELION processing pipeline, particles picked by DRPnet result in more “good” 2D class averages classes, (49 classes, Fig. 4c) compared to particles picked by RELION (32 classes, Fig. 4d). We also studied the angular distribution of the picked particles using angular coverage plots (Fig. 4e, f). In these plots, appearing views are indicated by dots on the 2D Hammer projection surface. The number of each view is proportional with size and color of corresponding dot. The angular coverage plots show that the distribution of DRPnet particle orientations (Fig. 4e, see also “Number of Views” in Table 3) were more distributed and numerous compared to those picked with RELION TBA (Fig. 4f). This result also agreed with the efficiency number E$_{\text {od}}$ [47] reported in Table 3, with DRPnet having a E$_{\text {od}}$ of 0.57 while RELION had E$_{\text {od}}$ of 0.51. Without imposing symmetry on the TRPV1 3D reconstructions (Table 3, indicated with parentheses), we note that DRPnet’s picked particles had improvements in angular coverage, which was further corroborated with improved E$_{\text {od}}$, as well as improved 3D reconstruction resolution generated with decreasing numbers of particles (Fig. 4g, solid lines) when comparing to the same measurements from RELION TBA. Upon imposing symmetry (C4) for the final TRPV1 3D reconstructions using approximately 20,000 particles (Fig. 4a, b), the resolution of 3D reconstructions from both DRPnet & RELION TBA particles were 3.9 Å (Table 3, and Fig. 4g). The C4 symmetrized 3D reconstructions also didn’t show noticeable differences (Fig. 7a, b) nor did their Fourier Shell Correlations (Fig. 7c). The particles picked by the fully automated DRPnet pipeline were able to generate similar resolutions in symmetrized 3D reconstructions compared to interactive RELION TBA but at much faster speeds. DRPnet testing time was approximately $\sim 6$ s/micrograph onNvidia GTX 1080 GPU with 8GB memory and SSD storage. Whereas manual template selection in RELION took approximately $\sim 30$ mins for 1000 particles. DRPnet also increased angular coverage (Fig. 4e) and increased good 2D class averages (Fig. 4c), resulting in improved efficiency (Table 3) and improved resolution without symmetry, even with low particle numbers (500) (Fig. 4g). These features are particularly helpful in structure determination when a dataset is lacking particle numbers or has an unknown symmetry.

**Table 3:** Summary table of results using DRPnet and RELION TBA (values in parentheses were obtained without imposing symmetry)

Dataset	EMPIAR-10005		EMPIAR-10061		EMPIAR-10017	
Program	DRPnet	RELION	DRPnet	RELION	DRPnet	RELION
# Picked particles	61,282	61,599	60,267	61,597	49,604	49,855
# Selected particles	23,147	22,830	49,582	49,570	41,914	40,669
# Angular views (in total 12,288)	2105	1907	3092	3000	3103	3034
	(4481)	(3697)	(8269)	(7968)	(10,038)	(9876)
Efficiency E$_{\text {od}}$	0.57	0.51	0.82	0.80	0.69	0.69
	(0.47)	(0.32)	(0.76)	(0.74)	(0.70)	(0.70)
Resolution (Å)	3.9	3.9	2.8	2.8	4.2	4.3
	(6.6)	(6.8)	(3.0)	(3.1)	(4.7)	(5.1)
Local resolution (Å)	3.6	3.7	2.8	2.8	3.9	4.0
	(6.7)	(6.9)	(3.5)	(3.7)	(5.8)	(6.0)

Next, we performed testing with two other datasets to verify that the proposed DRPnet particle picking network trained with TRPV1/EMPIAR-10005 could not only select particles with diversified shapes quickly, but also select particles with improved angular coverage correlating with a resulting higher resolution 3D reconstruction.

#### EMPIAR-10061 ($\beta$-galactosidase) testing

In the second test, we picked particles from 661 selected micrographs from the EMPIAR-10061 dataset of $\beta$-galactosidase. $\beta$-galactosidase has a less compact more elongated shape (dimensions 180 Å x 140 Å x 87 Å) as compared to TRPV1/EMPIAR-10005 used for training. This high resolution dataset was collected with minimal defocus and at high magnification (pixel size of 0.3185Å) on an Gatan K2 energy-filtered direct detector (size of $7676\times 7420$ pixels), which resulted in extremely low particle contrast (see Fig. 1a). To pick particles from this dataset using DRPnet, the detection levels ($\hbox {sigma} = 1$, $\hbox {threshold} = 1\times 0.01$) and scale factor of 1/8 were set as input parameters. DRPnet second classification CNN (Fig. 2c) was retrained with positive samples composed of subset of detected particles by the DRPnet FCRN network having high standard deviations ($\hbox {s} > 0.25$) and negative samples as a subset of detected particles having low standard deviation ($\hbox {s} < 0.25$). Note that the first CNN (FCRN detection network) was not retrained. Only the second CNN (refinement network) was retrained. Labeled training data for the second CNN was generated in an unsupervised manner, without need for any external annotation, by thresholding the output of the first CNN. DRPnet picked 60,267 particles from the EMPIAR-10061 micrographs (Table 3 and Fig. 5a). To obtain similar number of particles, for RELION TBA, we selected $\hbox {sigma} = 1.2$ and $\hbox {threshold} = 0.15$ and picked 61,597 particles (Table 3 and Fig. 5b). All subsequent jobs were performed in RELION 3.0, including extraction, classification and refinement. We used a binning factor of 2, a box size of $384\times 384$ pixels and a pixel size of 0.637 Å. During the 3D structure refinement step, we kept 49,592 and 49,570 good particles from DRPnet and RELION TBA, respectively (Table 3), and once again noted more 2D classes for DRPnet (Fig. 5c) versus RELION TBA (Fig. 5d). The reported resolution of the resulting 3D reconstructions were 2.8 Å for DRPnet and RELION (Table 3) using D2 symmetry. Although the 3D reconstruction FSC global resolutions were similar, Fig. 5e, f illustrate the angular distribution of particles picked from the EMPIAR-10061 dataset ($\beta$-galactosidase). The plots of angular coverage show that the particles of DRPnet (Fig. 5e, f) represented more views than particles of RELION TBA (Table 3). This result also agreed with an improved efficiency factor (E$_{\text {od}}$ in Table 3). To visualize all views picked by either DRPnet or RELION TBA, no symmetry (C1) was imposed and the corresponding C1 values are listed in parentheses in Table 3. DRPnet shows that more views were picked in C1, having improved E$_{\text {od}}$ and resolution than RELION TBA. Looking at the resolution imposing two-fold (D2) symmetry, DRPnet had E$_{\text {od}}$ of 0.82 while RELION had E$_{\text {od}}$ of 0.80. Finally, a Rosenthal and Henderson B-factor plot (Fig. 5g) shows that when the number of EMPIAR-10061 particles increase from 1200 to 38,000 particles, the 3D reconstruction from DRPnet’s particles yield higher resolutions compared to RELION TBA’s—both with and without symmetry.

#### EMPIAR-10017 ($\beta$-galactosidase) testing

After testing DRPnet particle picking on the EMPIAR-10005 (TRPV1) and EMPIAR-10061 ($\beta$-galactosidase) datasets, we conducted the third test on the all micrographs in the deposited EMPIAR-10017 ($\beta$-galactosidase) dataset. Note, these $\beta$-galactosidase protein particles are the same as the previous test, however with different data collection conditions. This dataset was collected on an older generation cryo-transmission electron microscope (FEI F30 Tecnai Polara) and a different direct detector (FEI Falcon II), having an image size of $4096\times 4096$ pixels and a pixel size of 1.77 Å. We set DRPnet to detect particles at picking level (sigma = 7, threshold = $2\times 7$), and selected training samples for classification at positive level (sigma = 16, threshold = $3\times 7.5$) and negative level (sigma = 12.5, threshold = $2\times 5$) to pick 49,604 particles (Table 3 and Fig. 6a). RELION TBA parameters were set to pick 49,855 particles (Table 3 and Fig. 6b). Those particles were extracted with a box size of $200\times 200$ pixels at original scale for 3D map reconstruction. Unlike the EMPIAR-10005 and EMPIAR-10061 datasets, whose ground truth particle sets consist of only selected good particles, the ground truth provided for the EMPIAR-10017 dataset consists of particles manually picked by an expert [11] and covers almost all the particles in the associated micrographs. Because of this fact, for the EMPIAR-10017 dataset, we were able to evaluate the reconstruction outcome of the picked particles, and directly evaluate their detection performance using Recall, Precision, and F-measure metrics. Table 4 shows that while RELION TBA results achieved recall, precision, and F-measure values of 73.4 %, 59.9 %, and 65.9 % respectively, the proposed DRPnet system achieved recall, precision, and F-measure values of 87.7 %, 71.1 %, and 78.5 %; a considerable improvement of more than 10% in each metric.

**Table 4:** Recall, precision and F-measure for the proposed automated particle picking pipeline evaluated on EMPIAR-10017 ($\beta$-galactosidase) versus other state-of-the-art pickers

Model training	Program	Version	# Picked particles	Recall (%)	Precision (%)	F-measure (%)
Pretrained	DRPnet	1	49,604	*87.7*	71.1	*78.5*
	RELION	3.0	49,855	73.4	59.9	65.9
	WARP	1.0.9	46,438	83.9	*73.6*	78.4
	TOPAZ	cryoSPARC 2.14.2	51,749	83.4	64.9	73.0
	crYOLO(*)	1.7.5	49,329	**90.9**	**74.8**	**82.1**
	DeepPicker	1	40,880	26.5	26.4	26.5
Trained from scratch with TRPV1 (EMPIAR-10005)	DRPnet	1	49,604	**87.7**	**71.1**	**78.5**
	RELION	3.0	49,855	73.4	59.9	65.9
	WARP	1.0.9	49,209	*85.0*	*69.7*	*76.6*
	TOPAZ	cryoSPARC 2.14.2	48,208	72.3	60.7	66.1
	crYOLO	1.7.5	49,742	59.2	48.2	53.1
	DeepPicker	1	40,205	23.3	23.6	23.5

The best scoring is indicated in bold and the second-best in italics

*The pretrained model of crYOLO made available by its developers was trained with a training set that included our test set while training sets for all other pickers did not include particles from the test set

With the total number of picked particles around 50,000 particles from each algorithm, using RELION’s 3D reconstruction pipeline, we performed 2D classification to identify particles sorting into well-defined 2D class averages. Visually, we selected only good 2D class averages for further 3D classification, selecting the best for 3D refinement and reconstruction. The number of good particles sorting into defined 2D class averages corresponding to either DRPnet’s pick or RELION TBA are 41,914 or 40,669, respectively (Table 3). Again we note the increased number of 2D classes output for DRPnet (Fig. 6c) versus RELION TBA (Fig. 6d). Our experiment shows that map generated from DRPnet-identified particles had a resolution of 4.2 Å while RELION structure had a resolution of 4.3 Å. Figure 6e, f illustrate the angular coverage of particles picked from dataset EMPIAR-10017 ($\beta$-galactosidase). These plots show that the particles of DRPnet improved angular coverage than particles from RELION TBA. With symmetry imposed (D2), Table 3 shows DRPnet covers 3103 views while RELION TBA has 3034 views. Without symmetry (C1), DRPnet increases to 162 views (see Table 3). Both DRPnet and RELION TBA’s particles had the same efficiency E$_{\text {od}}$ of 0.69 with two fold symmetry (D2), and 0.70 without symmetry (C1). The Rosenthal and Henderson B-factor plot in Fig. 6g shows that when the number of EMPIAR-10017 particles increase from 1200 to 38,400 particles, the resolution of DRPnet’s 3D reconstruction was improved compared to RELION TBA, especially in case of no symmetry (C1).

In Fig. 7, we display local and global (Fourier Shell Correlation cutoff of 0.143) 3D reconstruction resolution results for EMPIAR-10005 (TRPV1) in the first row (Fig. 7a–c), EMPIAR-10061 in the second row($\beta$-galactosidase) (Fig. 7d–f), and EMPIAR-10017 in the last row ($\beta$-galactosidase) (Fig. 7g–i). The 3D reconstructions generated from DRPnet- (left, Fig. 7a, d, g) and RELION- (middle, Fig. 7b, e, h) picked particles are rendered as isosurfaces, contoured at the same level using the UCSF Chimera visualization software [53]. The detailed differences of resolution between 3D reconstructions from DRPnet-picked particles and RELION TBA particles are shown using a local resolution-based color-scheme with blue representing areas with higher resolution, red with lower resolutions. DRPnet-picked 3D reconstructions show increased local-resolution compared to RELION TBA’s 3D reconstructions, indicating an overall improved particle orientation and alignment. The local resolution ranges of those maps for EMPIAR-10005 are 3.6–6.7 Å (by DRPnet) and 3.7–6.9 Å (by RELION TBA), for EMPIAR-10061 are 2.8–3.5 Å (by DRPnet) and 2.8–3.7 Å (by RELION TBA), and for EMPIAR-10017 are 3.9–5.8 Å (by DRPnet) and 4.0–6.0 Å (by RELION TBA). The masked FSC curves produced by RELION’s post-processing task are shown (Fig. 7c, f, i), with and without imposing symmetry, for both DRPnet-picked and RELION TBA’s particles. The DRPnet-picked 3D reconstruction Fourier Shell Correlation curve extends to a slightly higher resolution than RELION TBA’s reconstruction in all 3 datasets, both with and without imposing symmetry.

#### Comparison of particle picking networks

DRPnet’s performance was evaluated in terms of recall, precision, and F-measure [54], and compared to other previously reported state-of-the-art deep learning-based particle picking networks. Four networks were compared to DRPnet: WARP (version 1.0.9) [20] , TOPAZ (version 0.2.5 implemented in CryoSPARC 2.14.2) [19] , crYOLO (version 1.7.5) [55] , and DeepPicker (version 1) [18]. Table 4 shows these comparisons. These deep learning-based tools (especially WARP and TOPAZ) have become widely adopted in the cryoEM community to perform the particle picking task. For our first experiment, using their pretrained network models as downloaded from Github, each network was tested on the full EMPIAR-10017 ($\beta$-galactosidase) dataset (84 cryoEM micrographs) [50]. Recall, precision, and F-measure values were computed for each network and compared to DRPnet’s values (Table 4, “Pretrained” Model). As mentioned previously, DRPnet had a recall of 87.7%, precision of 71.1%, and F-measure of 78.5%. DRPnet performed better than all others tested, with the exception of crYOLO. Pretrained model of crYOLO [21] was previously trained on multiple datasets by its developers including EMPIAR-10017, thus crYOLO had an unfair advantage as this network had already seen this testing data. In our second experiment, each network was trained from scratch with EMPIAR-10005 (TRPV1) [36] using default training settings indicated by the developers, then tested on EMPIAR-10017 ($\beta$-galactosidase) dataset, and recall, precision, and F-measure values were compared. DRPnet outperformed all other networks as shown in (Table 4, “Trained with TRPV1” Model). This is true even for crYOLO, whose performance drastically drops when tested on unseen data. Although we used the network developers’ suggested settings for training, retraining the networks could suffer from inappropriate settings used and/or amount of training data used. When we retrained the networks from scratch on 1800 particles, the performance decreased. This performance decrease could be attributed to limited quantity of training data used when training from scratch. If DRPnet training from scratch is required, one benefit of DRPnet is less data quantities are required compared to other networks. Particle picking results for a sample image obtained using the pretrained and retrained networks listed in Table 4 are shown in the Additional file 1: Figures S1 and S2. Corresponding quantitative evaluation results are given in Table S1. Further sample results demonstrating the DRPnet’s particle picking performance on negatively stain datasets (ALDH7A1, BiFab OKT3) and on an additional cryoEM dataset (EMPIAR-10025 T20S proteasome cryoEM) are shown in Additional file 1: Figures S3 and S4, respectively. These results demonstrate generalization capabilities of DRPnet on unseen data.

## Conclusions

In summary, we propose a Deep Regression Picker Network (DRPnet) and successfully demonstrate the ability to pick particles on a multiple datasets of cryo-EM micrographs which is different from the training data (TRPV1,EMPIAR-10017)—different sized/shaped/separated particles, collected on different microscopes, using different cameras, with different background contrast (ie. negative stain)—and greatly reduced the time-consuming barrier of manual picking to generate a template (as in RELION’s template based autopicking). Our deep learning-based network is simple and effective for automatically picking particles from 2D cryo-EM micrographs, including those with low contrast, having a large particle box size, having differing particle shapes, handling particles that are clumped, using data collected with different cryo transmission electron microscopes, with different direct detection cameras, and different defocus ranges. We have also successfully used DRPnet to pick particles from negatively stained data (Additional file 1: Figure S3), which has been reported to be a challenge with other deep-learning based tools [19].

DRPnet has a cascade of two convolutional neural networks—the first for detection, followed by a second for classification. This cascade architecture provides flexibility to retrain either or both networks if desired. Compared to other deep learning tools for particle picking of cryoEM micrographs reported to date, this cascade strategy is unique. After the initial training with TRPV1, we found it was unnecessary to retrain DRPnet when applied to other cryoEM micrographs containing differently shaped particles in other datasets (e.g. $\beta$-galactosidase, EMPIAR-10061 & EMPIAR-10005). Without the need to retrain, automated particle picking using DRPnet to assist in 3D reconstruction of protein structures by single particle analysis can be realized. If desired, one can retrain the classification CNN—which may be useful for very low contrast images or images that have artifacts, such as ice contamination with similar dimensions to particles of interest, or if the data exhibits a different pattern or illumination.

Utilizing a pretrained network to pick particles significantly reduces time to obtain a particle dataset. DRPnet is very efficient for selecting a set of particles. For comparison, picking $\sim \,1000$ particles manually in our laboratory from the above EMPIAR datasets took about 30 min per dataset. With manually picking particles, there is also additional time spent running the manually picked 2D classification (10 min), selecting good 2D class average templates (1 min), as well as more time invested optimizing template-based autopicking parameters (10 min) and finally running the autopicking job (10 min or longer). With DRPnet, it takes about 6 s per micrograph to pick the particles, which results in significant time savings, as compared to generating a quality particle dataset in similar quantity via RELION’s workflow.

Considering the size of scaled input particles (64 x 64 pixels), the proposed network architecture has been kept simple to maintain an appropriate number of neural network parameters to avoid convergence issues and to achieve faster performance. The current network configurations are adequate for our picking pipeline and therefore implementing more complex architectures is unnecessary. Our results show that DRPnet architecture performs better than other more complex architectures (WARP uses UNet, crYOLO using YOLO), which as we show in Table 4 can be inhibitive for particle picking. Considering that we achieve this even with smaller training sets also emphasizes the effect of simpler architecture.

Investigating traditional computer science detection/classification evaluation metrics of recall, precision, and F-measure, we performed a comparison between popular deep learning-based particle picking networks (WARP, TOPAZ, crYOLO, DeepPicker)—both with and without model retraining. We note DRPnet had comparable or improved evaluation metrics compared to all programs attempted, with or without training—with the exception of crYOLO, which had been trained previously on our test dataset (EMPIAR-10017). While these supervised evaluations are very informative, many times, existing ground truth sets are lacking due to 1) low contrast particles not contained in the “expert’s” ground truth, or 2) the particles composing final 3D reconstruction not representing the total particles used for alignment, classification, and averaging. Specifically, in case of EMPIAR-10017 ($\beta$-galactosidase), although the ground truth was the most comprehensive, it still did not cover all particle patterns, thus orientations. This may be due to fluctuations in intensity level, which may effect the ground truth’s particle patterns. Cryo-electron microscopy images captured with low contrast (low defocus or minimal electron radiation exposure, therefore enhancing high resolution terms) likely have high resolution 3D protein structural information, and conversely, those captured with high contrast (high defocus or high electron radiation exposure, enhancing low resolution terms) contain low resolution information. Because images in EMPIAR-10017 ($\beta$-galactosidase) have different contrast levels, a true positive particle in a low contrast image can be a false positive pattern in high contrast image.

For the above reasons, the detection stage F-measure of 78.5% was not high, but it was the best result we could achieve with EMPIAR-10017 ($\beta$-galactosidase) data. We note however DRPnet was the best compared to deep-learning particle picking networks (WARP, TOPAZ, DeepPicker) that hadn’t been previously trained on this data, and the discrepancy becomes even wider upon model training from scratch. In addition to supervised detection evaluation metrics, we sought additional unsupervised metrics to guide our analysis even when complete ground truths are not available. Metrics we found useful include angular distributions plots, efficiency (E$_{\text {od}}$) values [47], Rosenthal and Henderson B-factor plots [48] with and without imposing symmetry, inspecting the 3D isosurface using local-resolution based color scheme, and finally reporting resolution using the gold standard Fourier Shell Correlation (correlation threshold of 0.143) [48].

We consistently obtained more 2D classes, and improved angular coverage. The corresponding 3D reconstructions had slightly higher global & local resolutions with DRPnet picked particles compared to RELION’s TBA particles. DRPnet excelled in particle picking with low contrast data sets (those collected on Gatan direct detectors using images collected close to focus). Plotting the angular distributions allows one to visually inspect the orientations (angles and rotations) of the picked particles and also their abundance. With DRPnet picked particles, a broader coverage of angular space is observed (less white background in the 2D Hammer projection surface) for all datasets tested. This finding is also corroborated by the efficiency metric, with DRPnet consistently showing improved E$_{\text {od}}$ values, indicating a more robust sampling of particle angular orientations in the final 3D reconstruction. Finally, visual inspection of the 3D reconstruction isosurface when displaying the variation in local resolution indicates improved particle alignment with DRPnet-picked versus RELION TBA’s 3D reconstructions, indicated by more blue (high resolution) and less red (lower resolution) isosurface. We conclude DRPnet, based on the concept of blob detection, can pick diversified patterns which usually results in wider particle orientation angle coverage, yielding improved resolution. This is different than the manually selected templates used in RELION TBA. Especially for low contrast (low defocus, high resolution) datasets, bias from the user picking manual particles is minimized when using DRPnet—not only are preferred high contrast particle views picked, but also other less obvious views with less contrast—yielding a particle set from DRPnet having more diverse angular orientations. If limited by either input particle quantity, unknown symmetry or inability to apply it (for example variable ligand binding stoichiometries, variable conformations or allostery), DRPnet may be particularly useful in picking particles that have improved angular orientation sampling leading to improved particle alignments. All these benefits may likely result in a 3D single particle reconstruction with improved final resolutions—both global and local. Future plans include implementation in an open-source framework (Pytorch, for example), reducing the barrier to DRPnet’s widespread adoption. Also, addition of powerful classification networks to our workflow, trained with multiple types of particles (e.g. heterogeneous populations of particles) would likely reduce the running time of our picking software while increasing its accuracy (reducing false positives such as noisy and/or low contrast particles).

Here, we present a deep regression-based particle picking network composed of a dual cascade of convolutional neural networks, entitled “DRPnet”. The advantages of automated particle picking with DRPnet are following: (1) the picking algorithm does not have to be, but can be, retrained, (2) the efficiency of automated particle picking compared to manual template generation, and (3) the ability of DRPnet to perform picking on low contrast cryoEM datasets (low defocus, high resolution) or data that is not complete (poor angular sampling). We compare DRPnet performance to popular, deep-learning based particle picking networks in terms of detection evaluation metrics, recall, precision, and F-measure, and show that is comparable or better with and without training the respective models. We also provide functional assessment metrics—higher quantities of good 2D class averages, better efficiency reflected in angular coverage plots, Rosenthal–Henderson plots, assessing global/local resolution—for cryoEM micrograph particle picking beyond traditional evaluation metrics. We envision these metrics will be useful to the cryoEM community when assessing the performance of future particle picking networks.

## Supplementary information

**Additional file 1:** Supplemental materials.

## Data Availability

The datasets used for our experiments were downloaded from EMPIAR (https://www.ebi.ac.uk/pdbe/emdb/empiar/). DRPnet is freely available on GitHub (https://github.com/emcoregit/DRPnet).

## Abbreviations

CryoEM: Cryo-electron microscopy
TBA: Template based autopicking
TEM: Transmission electron microscope
DRPnet: Deep regression picker network
FCRN: Fully convolutional regression network
CNN: Convolutional neural network
SNR: Signal-to-noise ratio
FSC: Fourier shell correlation
