# Supplementary material for: DRPnet: automated particle picking in cryo-electron micrographs using deep regression
Source: BMC Bioinformatics. 2021 Feb 8;22:55. doi: 10.1186/s12859-020-03948-x (PMC7869254; doi:10.1186/s12859-020-03948-x)
Supplement: Supplementary file 1 — Additional file 1: Supplemental materials. [file 12859_2020_3948_MOESM1_ESM.pdf]

Supplementary Materials for "DRPnet - Automated Particle Picking in Cryo-Electron Micrographs using Deep Regression"

Nguyen P. Nguyen[1&5] Ilker Ersoy[2] Jacob Gotberg[3] Filiz Bunyak[1] Tommi White[4&5]
[1] *Department of Electrical Engineering and Computer Science, University of Missouri, Columbia, MO, United States*
[2] *Institute for Data Science and Informatics, University of Missouri, Columbia, MO, United States*
[3] *Research Computing Support Services, University of Missouri, Columbia, MO, United States*
[4] *Department of Biochemistry, University of Missouri, Columbia, MO, United States and*
[5] *Electron Microscopy Core, University of Missouri, Columbia, MO, United States*

Particle picking performance on a single cryoEM micrograph using pretrained or newly trained models

To provide detection examples of deep-learning based particle picking networks, a single cryoEM micrograph was selected from EMPIAR-10017 β-galactosidase and particles were picked (yellow circle, Figures S1 & S2) and compared to the ground truth particle location (green dot). These testing experiments were performed using the pretrained model (Figure S1), and also a newly trained model (Figures S2), which was trained on a subset of EMPIAR-10005 TRPV1. The recall, precision and F-measure was then calculated based on the test detection results of this single image (Table S1). This analysis was performed using WARP [1], TOPAZ [2], crYOLO [3], and DeepPicker [4]. The results are compared to DRPnet (trained from scratch only once with the same subset of TRPV1) and to the RELION Template Based Autopicking. As also shown in the main text, DRPnet outperforms all other pretrained deep-learning based particle picking networks on this single EMPIAR-10017 (β-galactosidase) cryoEM micrograph (Table S1, upper) - with the exception of crYOLO, which the model was pretrained with this dataset. Upon training the deep learning-based particle picking networks from scratch with the same subset of EMPIAR-10005 TRPV1 (used to train DRPnet once), DRPnet clearly outperforms in terms of traditional evaluation metrics ((Table S1, lower). Visual inspection of Table S1's results is available in Figures S1 & S2.

TABLE S1: Recall, Precision & F-measure calculated from particle picking on a single EMPIAR-10017 (β-galactosidase) cryoEM micrograph

Model	Network	Recall	Precision	F-measure
Pretrained models	DRPnet	**92%**	**90%**	**91%**
	RELION	74%	83%	78%
	WARP	<u>88%</u>	<u>87%</u>	<u>88%</u>
	TOPAZ	84%	80%	82%
	crYOLO(*)	96%	89%	93%
	DeepPicker	29%	30%	29%
Models trained from scratch	DRPnet	**92%**	**90%**	**91%**
	RELION	74%	83%	78%
	WARP	<u>90%</u>	<u>87%</u>	<u>88%</u>
	TOPAZ	75%	71%	73%
	crYOLO	63%	53%	59%
	DeepPicker	28%	29%	28%

(*) The pretrained model of crYOLO is available and was previously trained by its developers with a training set that included our test set (EMPIAR-10017 β-galactosidase), while the training sets used for generating the pretrained model for all other pickers did not include particles from the test set.

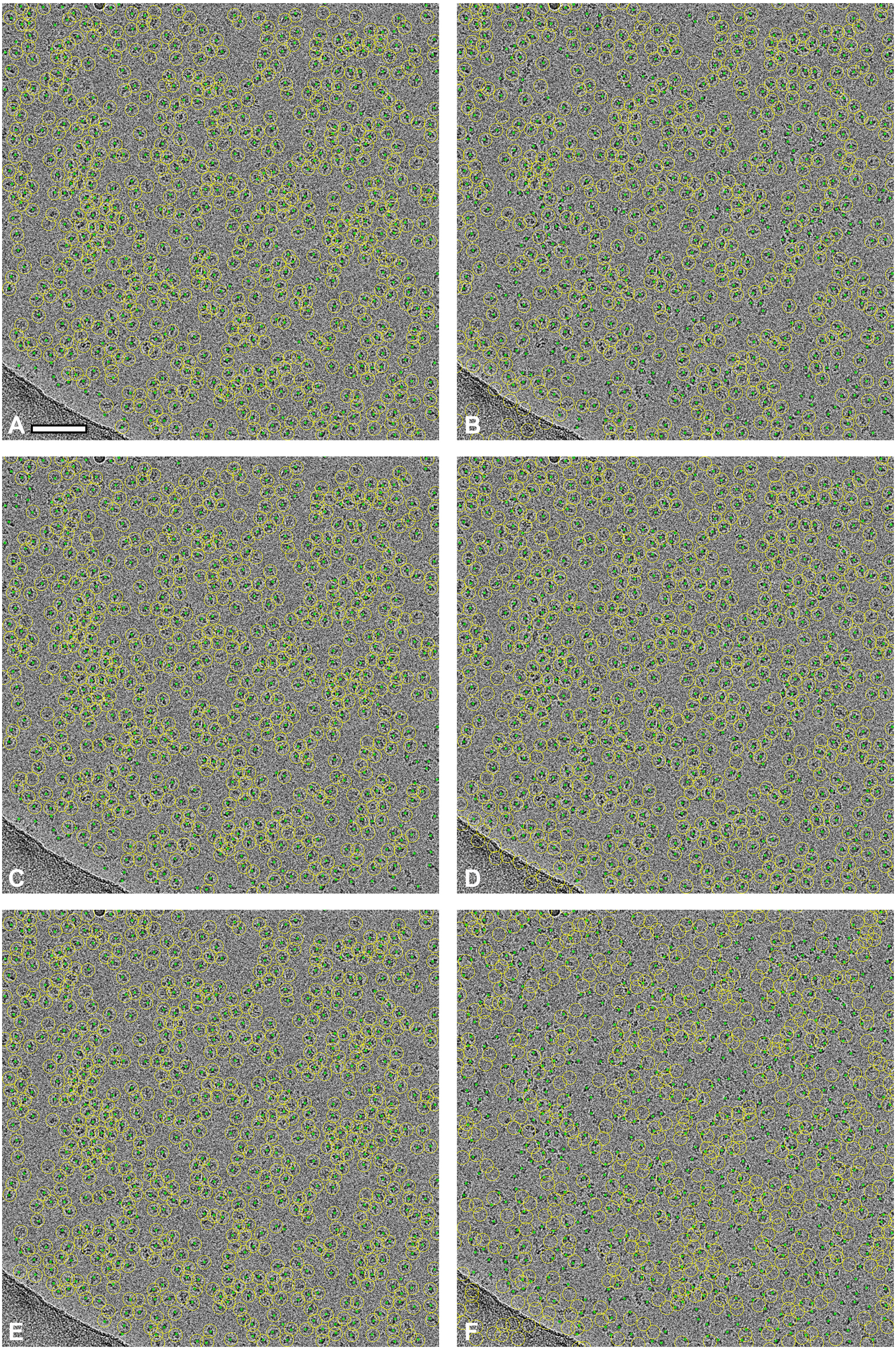

FIG. S1: Particle picking on a single cryoEM micrograph from EMPIAR-10017 β-galactosidase using pretrained models of DRPnet (A), WARP (C), TOPAZ (D), crYOLO (E) and DeepPicker (F). RELION's template-based autopicking (B) was also compared. Yellow circles represent detected particles, green dots display the ground truth centers. Scale is 885 Å.

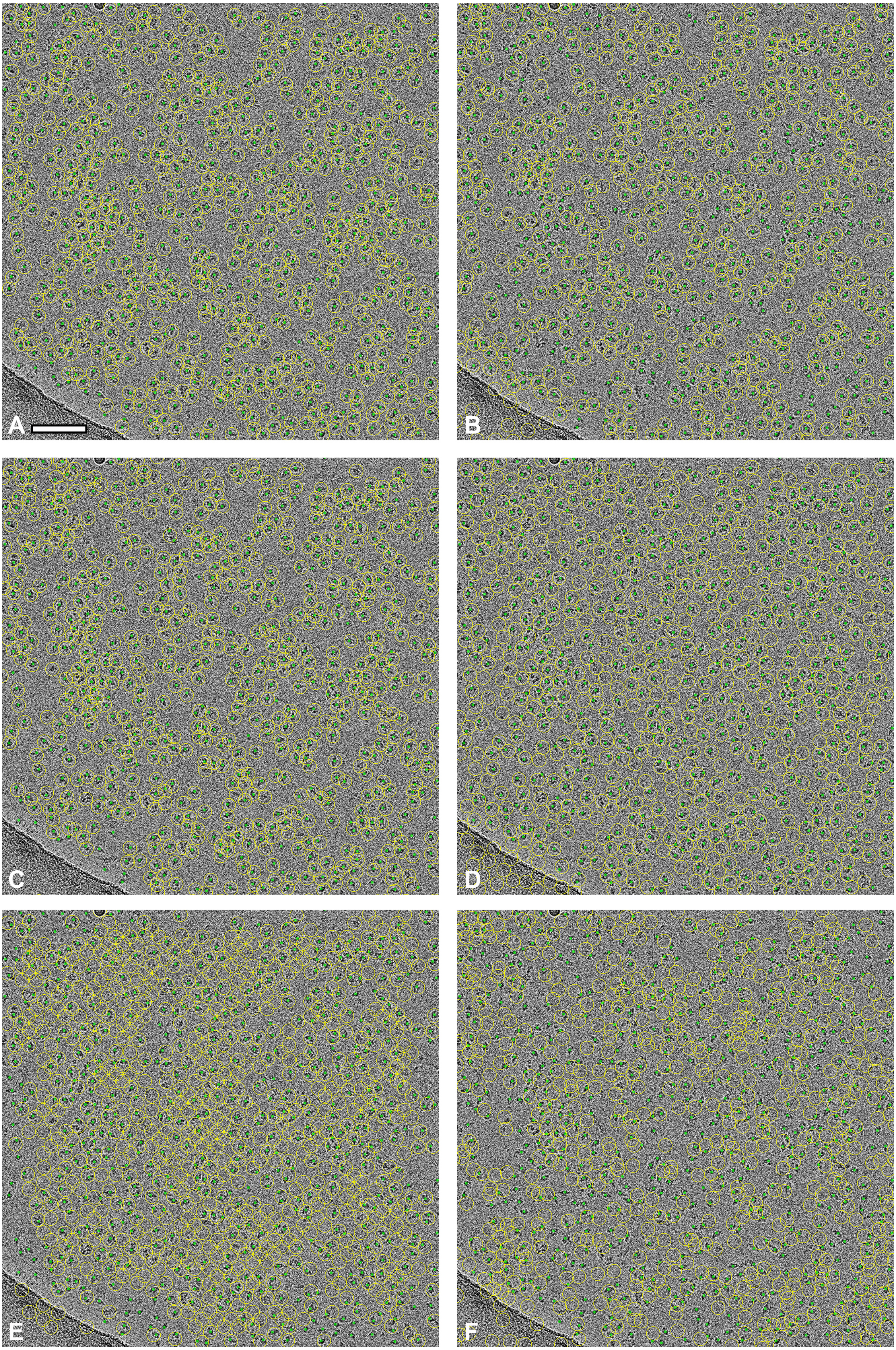

FIG. S2: Particle picking on a single cryoEM micrograph from EMPIAR-10017 β-galactosidase using TRPV1-trained models of DRPnet (A), WARP (C), TOPAZ (D), crYOLO (E) and DeepPicker (F). RELION's template-based autopicking (B) was also compared. Yellow circles represent detected particles, green dots display the ground truth centers. Scale is 885 Å.

DRPnet on other datasets: negative stain data and EMPIAR-10025

DRPnet relies on the blob detection concept to recognize protein particles in cryo-electron micrographs. To demonstrate the ability of DRPnet to recognize particles of various shapes, sizes and embedding media (ex. heavy metal stains), we used multiple datasets to display DRPnet's versatility. DRPnet is able to produce suitable particle datasets using negatively stained micrographs, which display an inversion of contrast (dark background, white particles) when compared to cryo-electron micrographs (light background, dark particles). The background of negative stain images is noisy with wide variations in transmission/illumination, usually due to changing stain thickness. DRPnet is also able to pick particles having an elongated dimension and/or hollow centers, which was previously reported challenge for other particle picking packages [5].

To show the effectiveness of DRPnet in picking on negatively stained datasets, we chose two examples: aldehyde dehydrogenase 7A1 (ALDH7A1) and a Fab fragment OKT3. These datasets were collected by University of Missouri Electron Microscopy Core users using uranyl formate negative stain. Negatively stained micrographs were collected on either the FEI Tecnai F30 Twin TEM equipped with Gatan Ultrascan 4000 (ALDH7A1) or the JEOL JEM 1400 equipped with a Gatan Ultrascan 1000 (OKT3). Using 20 negatively stained micrographs of the apo ALDH7A1 dataset [6], DRPnet detected 5,727 particles (Figure S3a-b). In the apo form, ALDH7A1 is present as a mostly as a dimer (100 Å x 45 Å), which is a quarter the size of β-galactosidase (180 Angstrom x 140 Angstrom) as described in the main text. Another negative stain dataset containing self associating "biFab" fragments, OKT3 [7], was used. BiFab OKT3 is composed of two OKT3 Fab fragments (60 Å x 30 Å) that displays an undetermined flexible association, with each Fab in the biFab particle showing a range of conformations. In the elongated state, being two Fabs end to end composes the longest conformation, with a length of 120 Angstroms. DRPnet picked 467 OKT3 Bifab particles (Figure S3c-d) from 4 micrographs. This molecule displays an elongated conformation in negative stain. Despite the particle being quite small, DRPnet is able to identify these particles well in negative stain. In addition to being able to pick β-galactosidase and TRPV1 particles in cryoEM micrographs, DRPnet is able to identify smaller particles, ALDH7A1 and OKT3 BiFab, in negatively stain micrographs.

In the final test, we performed DRPnet particle picking on cryoEM micrographs of T20S proteasome (EMPIAR-10025) [8] to test its ability to select particles displaying different shapes in varying orientation. The 20S proteasome (750 kDa)is a cylindrically-shaped molecule with a hollow center, displaying both a circular shape (120 Angstroms diameter) in certain orientations and a rectangular shape (150 Angstroms) in others. From 29 micrographs, DRPnet picked 19,558 particles as illustrated in Figure S4, demonstrating that DRPnet is capable of picking particles of many different shapes, sizes and densities.

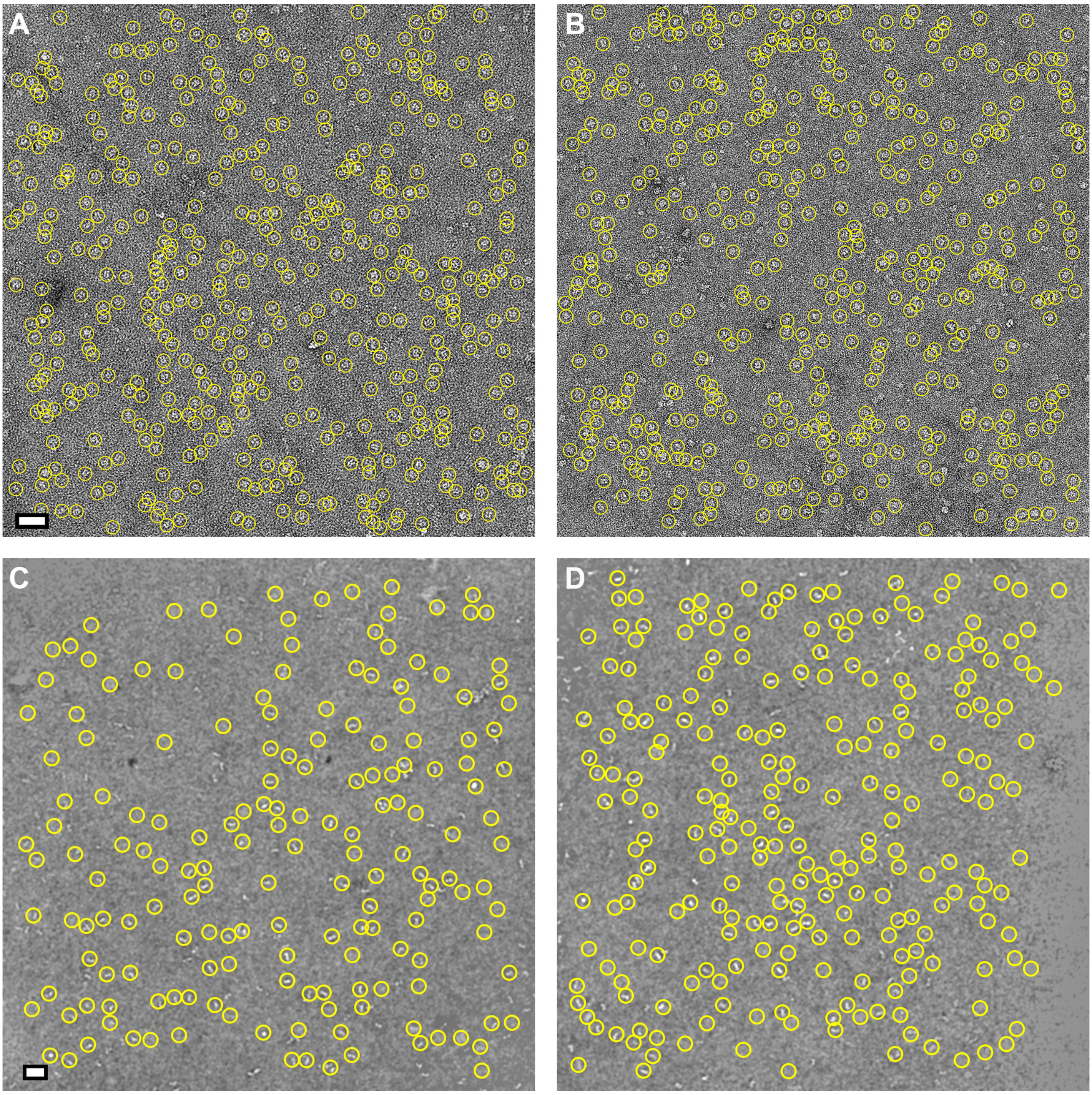

FIG. S3: DRPnet's test experiments on negatively stain datasets: ALDH7A1 (A-B) or BiFab OKT3 (C-D). Yellow circles identify picked protein particles (white densities) embedded in an uranyl formate negative stain (0.75%). Scale is 500 Å.

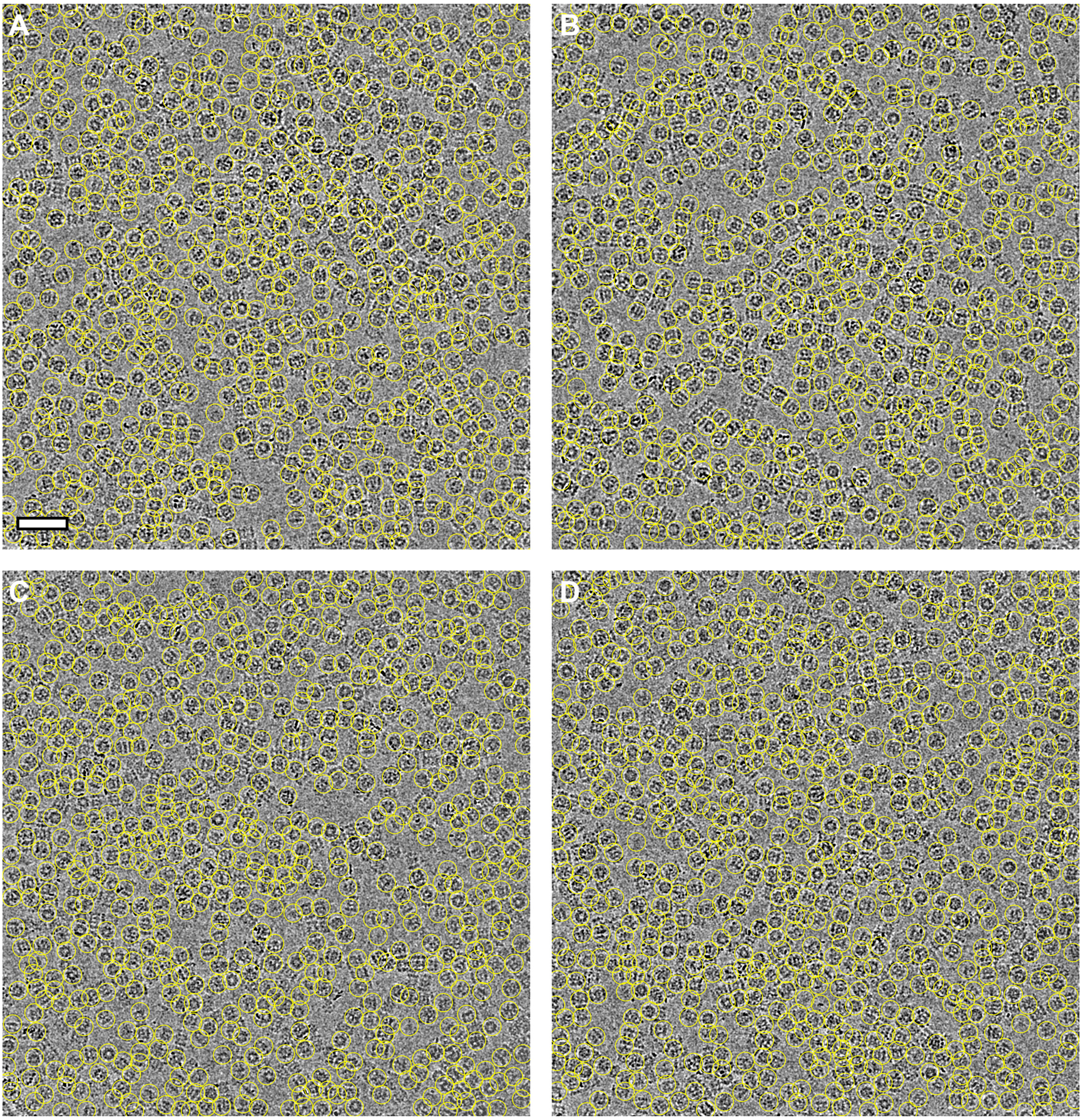

FIG. S4: DRPnet's particle picking on EMPIAR-10025. Four example cryoEM micrographs (A-D) of DRPnet particle picking from the T20S proteasome cryoEM dataset (scale is 500 Å). Yellow circles identify picked protein particles (dark densities) embedded in vitreous ice.

References

[1] Tegunov, D., Cramer, P.: Real-time cryo-EM data pre-processing with warp (2018). doi:10.1101/338558

[2] Bepler, T., Morin, A., Noble, A.J., Brasch, J., Shapiro, L., Berger, B.: Positive-unlabeled convolutional neural networks for particle picking in cryo-electron micrographs. Res Comput Mol Biol **10812**, 245–247 (2018)

[3] Wagner, T., Merino, F., Stabrin, M., Moriya, T., Antoni, C., Apelbaum, A., Hagel, P., Sitsel, O., Raisch, T., Prumbaum, D., Quentin, D., Roderer, D., Tacke, S., Siebolds, B., Schubert, E., Shaikh, T.R., Lill, P., Gatsogiannis, C., Raunser, S.: SPHIRE-crYOLO is a fast and accurate fully automated particle picker for cryo-EM. Communications Biology **2**(1), 1–13 (2019). doi:10.1038/s42003-019-0437-z

[4] Wang, F., Gong, H., Liu, G., Li, M., Yan, C., Xia, T., Li, X., Zeng, J.: Deeppicker: A deep learning approach for fully automated particle picking in cryo-EM. Journal of Structural Biology **195**(3), 325–36 (2016). doi:10.1016/j.jsb.2016.07.006

[5] Heimowitz, A., Anden, J., Singer, A.: Apple picker: Automatic particle picking, a low-effort cryo-EM framework. J Struct Biol **204**(2), 215–227 (2018). doi:10.1016/j.jsb.2018.08.012

[6] Korasick, D.A., Campbell, A.C., Christgen, S.L., Chakravarthy, S., White, T.A., Becker, D.F., Tanner, J.J.: Redox modulation of oligomeric state in proline utilization A. Biophys J **114**(12), 2833–2843 (2018). doi:10.1016/j.bpj.2018.04.046

[7] Pages, D.G., Nelson, A.D., White, T.A., Schrum, A.G.: Bivalent anti-cd3 fab dimers drive t cells into a highly efficient fratricide with potential clinical application. The Journal of Immunology **202**(1 Supplement), 68–186818 (2019). https://www.jimmunol.org/content

[8] Campbell, M.G., Veesler, D., Cheng, A., Potter, C.S., Carragher, B.: 2.8 Å resolution reconstruction of the *Thermoplasma acidophilum* 20S proteasome using cryo-electron microscopy. eLife **4**, 06380 (2015). doi:10.7554/eLife.06380
